# Comprehensive Molecular Analyses of an SLC Family-Based Model in Stomach Adenocarcinoma

**DOI:** 10.3389/pore.2022.1610610

**Published:** 2022-10-13

**Authors:** Tao Yu, Shao-kun Yu, Kai-hua Lu

**Affiliations:** Department of Oncology, First Affiliated Hospital, Nanjing Medical University, Nanjing, China

**Keywords:** immunotherapy, prognosis, STAD, solute carrier, amino acid

## Abstract

**Background:** Solute carrier (SLC) family members are crucial in transporting amino acids across membranes. Amino acids are indispensable for both cancer and immune cells. However, the clinical significance of amino acid transporting SLC members in stomach adenocarcinoma (STAD) remains unclear. This study aimed to develop an SLC family-based model to predict the prognosis and the response of STAD patients to immunotherapy.

**Methods:** A total of 1239 tumor cases were obtained from online databases. The training set (*n* = 371) consisted of RNA sequencing profiles obtained from The Cancer Genome Atlas (TCGA), while those from Gene Expression Omnibus (GEO) were used as the test set. Subsequently, the clinical characteristics and immune profiles were investigated, and potential immunotherapy response prediction values of the model were assessed.

**Results:** Based on the TCGA cohort, an SLC family-based model was developed using multivariate Cox analysis. All tumor cases were stratified into high- and low-risk groups considering the SLC model. High-risk patients had a worse overall survival (OS) than low-risk patients, consistent with the results of GEO cohorts. Comprehensive analyses revealed that the high-risk group was correlated with aggressiveness-related pathways, whereas the low-risk group had better T helper cell infiltration and stronger immunotherapy response. Compared to the high-risk group, the low-risk group presented increased PD-L1 and tumor mutation burden.

**Conclusion:** This SLC family-based model has the potential to predict the prognosis and immunotherapy outcomes of STAD patients. The survival of patients in the low-risk group was greatly prolonged, and the patients may benefit more from immunotherapy.

## Introduction

With about 762,000 deaths in 2020, stomach cancer is the fifth most common cancer worldwide [1]. The majority of stomach cancers are advanced at diagnosis, and the 5-year survival rate remains poor despite improved medical and surgical treatments. Immune checkpoint inhibitor (ICI) therapy has revealed promising outcomes in the CheckMate 649 clinical trial [2]. Thus, the US Food and Drug Administration (USFDA) has granted regulatory approval for using nivolumab in the first-line treatment of advanced or metastatic stomach cancer [2]. The efficacy of ICI therapy in suppressing programmed cell death-1 (PD-1) or programmed cell death ligand-1 (PD-L1) to enhance T cell functions is significantly correlated with host immune systems and the tumor microenvironment [3].

Amino acid metabolism has a crucial role in tumor immunity and affects the prognosis of ICI therapy. Glutamine and branched-chain amino acids (BCAAs, namely leucine, isoleucine, and valine) could serve as an alternative fuel to meet the nutrition demand of cancer cells by transforming into intermediate products of the tricarboxylic acid cycle [4, 5]. For example, glutathione, made from the amino acids glycine, cysteine, and glutamic acid, is crucial for antagonizing high levels of reactive oxygen species (ROS) originating from rapid proliferation and for maintaining the redox balance of cancer cells [6]. Similarly, highly proliferative immune cells depend on amino acid metabolism for biosynthesis. Furthermore, arginine contributes to the synthesis of T cell receptor CD3zeta chain and the generation of central memory T cells [7, 8]. Glutamine deficiency blocks T cell expansion, cytokine production, and helper T cell 1 (Th1) differentiation [9, 10]. Leucine, arginine, and glutamine are necessary for natural killer (NK) cell proliferation as these can activate mTOR signaling [11]. Methionine is crucial for generating S-adenosylmethionine (SAM) and sustaining methylation of histone and RNA [12]. In brief, the unlimited uptake of amino acids by cancer cells not only promotes tumor progression but also affects immune cell activation [8, 13]. Moreover, accumulated metabolites of amino acids like kynurenine (Kyn) could also impair the functions of T cells, NK, and dendritic cells (DCs) [14, 15]. However, efficient transportation of exogenous amino acids into the nucleus is necessary for their utilization. The transporter system, composed of the solute carrier (SLC) superfamily, is responsible for amino acid transportation across the plasma membrane. The SLC superfamily comprises over 400 transporters regulating the import and export of a wide range of metabolites [16]. More than 60 SLC transporters have been identified that are involved in amino acid transportation [17]. SLC1A5 and SLC7A5 are upregulated in several cancers depending on glutaminolysis. SLC1A5 and SLC7A5 have increased cell death resistance by inhibiting caspases and sustaining proliferative signaling *via* mTOR activation [18]. SLC7A11 has been overexpressed in most cancer types, including lung cancer, breast cancer, and ovarian cancer to promote tumor growth by suppressing ferroptosis [19]. However, the histology type of cancer affects the function of SLC proteins. For example, SLC1A5, which promotes the proliferation of basal-like triple-negative breast cancer, showed no impact on the growth of other breast cancer subtypes [20]. Notably, tumor cells with higher expression of SLCs absorbed more amino acids, resulting in immune cell inactivation and immune evasion [21]. Expression of SLC members like MCT1-4 was different in tumors with different immune subtypes such as the wound healing type, the inflammatory type, and the lymphocyte depleted type, suggesting the SLC family may be associated with tumor immunity [22].

In this study, we aimed to develop an SLC family-based model to predict the prognosis of stomach adenocarcinoma (STAD) and perform comprehensive analyses to verify the ICI therapy response prediction value of this model.

## Materials and Methods

### Patients and Datasets

RNA sequencing (RNA-seq) data of 371 STAD samples and their clinical information were downloaded from The Cancer Genome Atlas (TCGA) database (https://portal.gdc.cancer.gov/), while the RNA-seq data of 868 STAD samples (GSE84437, *n* = 433; GSE62254, *n* = 300; GSE183136, *n* = 135) and the corresponding survival information were downloaded from the Gene Expression Omnibus (GEO) database (https://www.ncbi.nlm.nih.gov/geo/). Their clinical information was collected in Supplementary Table S1.

### Model Construction and Validation

First, a univariate Cox regression analysis was performed to investigate the correlation between the gene expression value of SLC family members and the overall survival (OS) of patients with STAD. Subsequently, a stepwise multivariate Cox regression analysis was performed with the genes that significantly affect OS to develop a formula for the risk score. Patients were classified into high- and low-risk groups based on the risk score. Moreover, the prognostic power of the risk score was evaluated using Kaplan-Meier (K-M) survival curves with both TCGA and GEO cohort data. Furthermore, univariate and multivariate Cox regression analyses were performed to validate the independent prognostic value of the risk score.

### Gene Set Enrichment Analysis and Cancer Immunity-Related Analysis

To explore the functional signaling pathway, GSEA was performed. Gene sets with |normalized enrichment score (NES)|>1 more than 1, *p* < 0.05, and false discovery rate (FDR) <0.25 were considered to be significantly enriched [23].

Cell-type Identification by Estimating Relative Subsets of RNA Transcripts (CIBERSORT) algorithm was used to estimate the relative proportion of 22 immune cell types in the groups. Single sample GSEA (ssGSEA) was performed to further define the immune and molecular functions and compare the scores of the groups.

### Tumor Immune Dysfunction and Exclusion Analysis

The TIDE score, T cell dysfunction score, and T cell exclusion score of patients with STAD from the TCGA dataset were computed online (http://tide.dfci.harvard.edu) after uploading the transcriptome profiles. T cell dysfunction and T cell exclusion are the two primary mechanisms to model tumor immune evasion. TIDE integrates the expression signatures of T cell dysfunction and T cell exclusion, two primary mechanisms of immune evasion, to predict ICI therapy response [24].

### Statistical Analysis

All analyses were performed using R software (version 4.1.1, www.r-project.org) and codes are uploaded as Supplementary Datasheet S1. The independent t-test was performed to compare continuous values between the two groups, while the χ^2^ test was performed to examine categorical data. Spearman’s analysis was utilized to check the correlation between the groups. K-M survival analysis was utilized for univariate survival analysis, while the Cox regression model was utilized for multivariate survival analysis. Results with two-sides of *p* < 0.05 were considered statistically significant, providing credibility for the data analysis.

## Results

### Prognosis Values of SLC Family Genes in STAD

**FIGURE 1:**
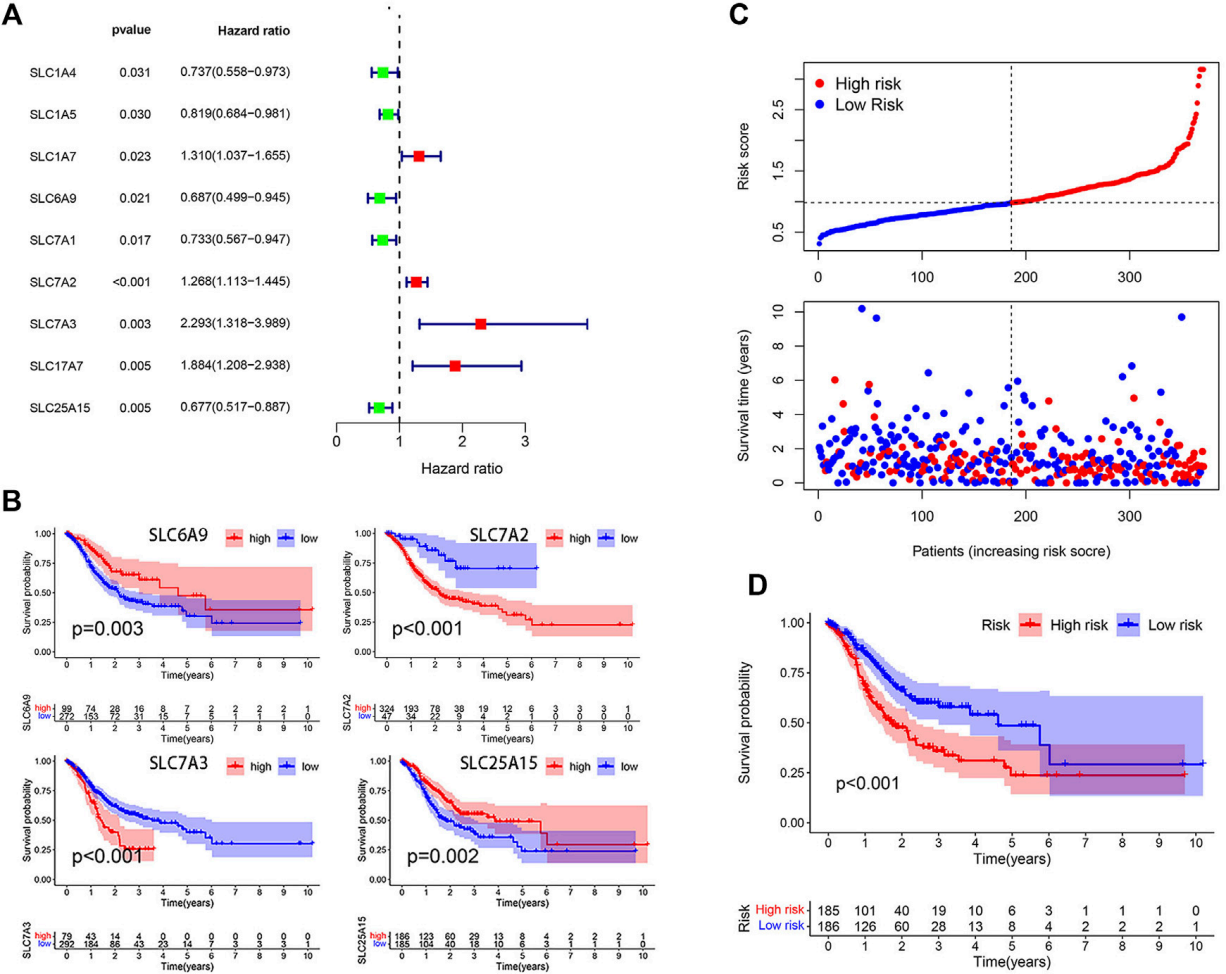
Model construction on the basis of TCGA database. **(A)** Forest plot of 9 SLC genes significantly affecting STAD prognosis. **(B)** K-M curves of 4 model-forming genes (cutoff values: SLC6A9: 1.2744, SLC7A2: 0.6353, SLC7A3: 1.3351, SLC25A15: 0.9835). **(C)** The distribution of risk score and survival status. **(D)** K-M analysis of two risk groups.

A total of 58 amino acid transporter-related SLC family genes were enrolled in this study. Univariate Cox analysis was used to evaluate the correlation between gene expression and OS (Table 1). Nine genes were found to be significantly associated with OS. SLC1A7, SLC7A2, SLC7A3, and SLC17A7 contributed to the poorer OS with a hazard ratio (HR) of more than 1. On the contrary, HRs of SLC1A4, SLC1A5, SLC6A9, SLC7A1, and SLC25A15 were less than 1 (Figure 1A). Subsequently, a stepwise multivariate Cox regression analysis was performed on nine genes, and four genes were ultimately confirmed to be significantly associated with prognosis (SLC6A9, SLC7A2, SLC7A3, and SLC25A15) (Supplementary Table S2). We also performed K-M analysis to identify the prediction potential of these genes and utilized the “surv_cutpoint” function to automatically determine optimal cutoff values (SLC6A9: 1.2744, SLC7A2: 0.6353, SLC7A3: 1.3351, SLC25A15: 0.9835). Results showed that SLC6A9 and SLC25A15 were positively correlated with OS, while SLC7A2 and SLC7A3 showed a negative correlation (Figure 1B). Consequently, a risk score formula was constructed, “risk score = 0.2384 * SLC7A2 expression + 0.4544 * SLC7A3 expression − 0.3645 * SLC6A9 expression − 0.3365 * SLC25A15 expression.” Tumor samples had significantly higher risk scores than normal samples (Supplementary Figure S1).

**TABLE 1:** Univariate Cox analysis of SLC family genes in the TCGA Cohort.

Gene	HR	HR.95 L	HR.95H	*p*-value	Predominant substrate (s)
SLC1A1	1.0033	0.8541	1.1786	0.9680	Glu, Asp, and Cys
SLC1A2	1.0742	0.7261	1.5892	0.7202	Glu and Asp
SLC1A3	1.0491	0.8694	1.2660	0.6168	Glu and Asp
SLC1A4	0.7366	0.5578	0.9728	0.0312	Ala and Ser
SLC1A5	0.8192	0.6843	0.9809	0.0300	Asp, Cys, and Gln
SLC1A6	1.1644	0.5441	2.4917	0.6950	Glu and Asp
SLC1A7	1.3100	1.0371	1.6546	0.0234	Glu and Asp
SLC3A1	1.0255	0.9042	1.1630	0.6953	—
SLC3A2	0.7840	0.5941	1.0347	0.0857	—
SLC6A5	2.6031	0.8732	7.7604	0.0861	Gly
SLC6A7	0.9110	0.5401	1.5365	0.7267	Pro
SLC6A9	0.6869	0.4993	0.9450	0.0210	Gly
SLC6A14	0.9276	0.8259	1.0418	0.2045	NAAs and CAAs
SLC6A15	1.5718	0.9149	2.7006	0.1015	BCAAs
SLC6A17	1.7533	0.8675	3.5436	0.1178	NAAs
SLC6A18	1.1455	0.6987	1.8778	0.5902	Gly
SLC6A19	1.0481	0.9112	1.2056	0.5109	NAAS
SLC6A20	1.0114	0.8630	1.1853	0.8884	Pro and Hyp
SLC7A1	0.7326	0.5669	0.9467	0.0174	CAAs
SLC7A2	1.2677	1.1125	1.4446	0.0004	CAAs
SLC7A3	2.2934	1.3185	3.9892	0.0033	CAAs
SLC7A5	0.9541	0.8285	1.0987	0.5141	LNAAs
SLC7A6	0.9223	0.6617	1.2855	0.6329	CAAs and LNAAs
SLC7A7	1.1886	0.9591	1.4730	0.1145	Cationic and NAAs
SLC7A8	0.9352	0.7639	1.1450	0.5167	LNAAs
SLC7A9	0.9593	0.8369	1.0997	0.5512	CAAs, cystine, Cys, and NAAs
SLC7A10	1.0432	0.8339	1.3049	0.7112	Small NAAs
SLC7A11	0.8312	0.6735	1.0259	0.0852	Glu and Cys
SLC7A13	1.8139	0.3446	9.5490	0.4822	Asp, Glu, and Cys
SLC7A14	1.4362	0.9356	2.2045	0.0978	CAAs
SLC15A3	0.9199	0.7602	1.1133	0.3913	His
SLC15A4	1.1369	0.7461	1.7323	0.5506	His
SLC16A10	1.0137	0.8039	1.2781	0.9087	Phe, Tyr, and Trp
SLC17A6	2.1357	0.8799	5.1837	0.0935	Glu
SLC17A7	1.8840	1.2081	2.9381	0.0052	Glu
SLC17A8	1.1681	0.7258	1.8800	0.5221	Glu
SLC25A2	0.9930	0.2406	4.0979	0.9922	Orn, Cit, Arg, and His
SLC25A12	0.8226	0.6182	1.0945	0.1802	Glu and Asp
SLC25A13	1.1190	0.8722	1.4357	0.3765	Asp and Glu
SLC25A15	0.6770	0.5168	0.8867	0.0046	Orn and Cit
SLC25A18	1.4740	0.8059	2.6962	0.2079	Glu
SLC25A22	0.8648	0.7090	1.0550	0.1520	Glu
SLC25A38	0.9138	0.6513	1.2820	0.6017	Gly
SLC32A1	3.9013	0.9825	15.4917	0.0530	Gly and GABA
SLC36A1	1.2430	0.9066	1.7042	0.1768	GABA
SLC36A2	1.4862	0.4443	4.9721	0.5201	Pro and Gly
SLC36A4	1.1966	0.9175	1.5604	0.1853	Pro and Trp
SLC38A1	0.9725	0.7976	1.1857	0.7829	Gln
SLC38A2	1.1071	0.8505	1.4410	0.4495	Gln
SLC38A3	1.1067	0.8303	1.4751	0.4894	Gln
SLC38A4	1.2379	0.9410	1.6284	0.1272	Gln and Arg
SLC38A5	1.1186	0.9712	1.2884	0.1200	Gln
SLC38A7	1.0525	0.7205	1.5373	0.7913	Gln and Ala
SLC38A8	1.1968	0.5609	2.5539	0.6422	Gln and Ala
SLC38A9	0.9845	0.6315	1.5348	0.9451	Arg and Leu
SLC38A10	0.9905	0.6933	1.4149	0.9580	Gln and Ala
SLC43A1	1.1698	0.9503	1.4400	0.1390	BCAAs
SLC43A2	1.0839	0.8547	1.3746	0.5064	BCAAs

**FIGURE 2:**
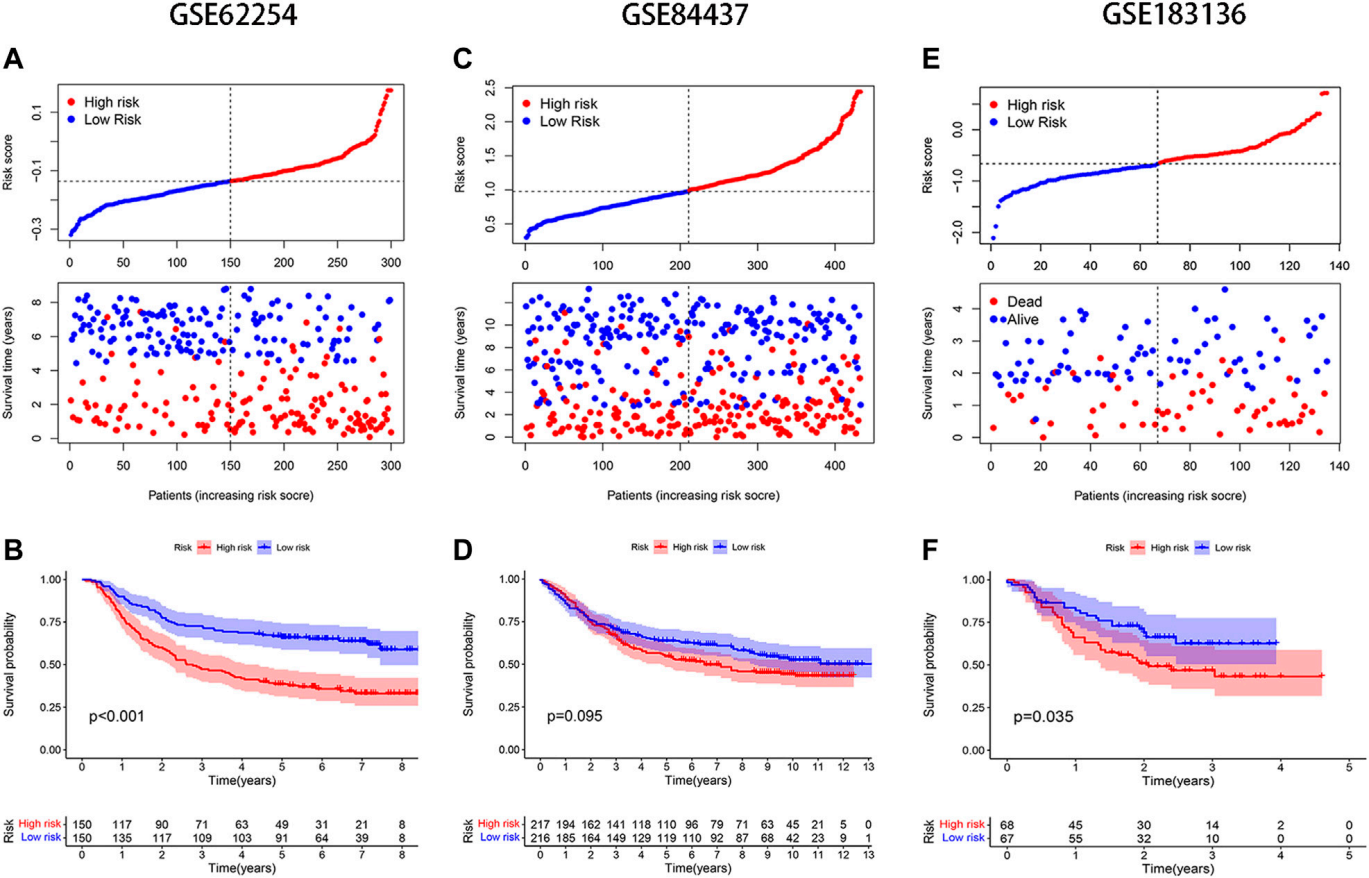
Validation of the model in different GEO datasets. Distribution of risk score and survival status: **(A)** GSE62254, **(C)** GSE84437, **(E)** GSE183136; K-M analysis of GEO databases: **(B)** GSE62254, **(D)** GSE84437, **(F)** GSE183136.

The median risk score was selected as a cut-off to divide all patients into the high- and low-risk groups. Univariate Cox regression analysis showed that age (≥65), TNM stage, T stage, lymphatic metastasis, and risk score were significantly correlated with the prognosis of STAD. Multivariate Cox regression analysis confirmed the independent prognostic value of the risk score (HR = 1.5623, 95% CI: 1.3210–1.8478, *p* < 0.0001) after adjustment of other clinicopathologic factors (Table 2). K-M analysis demonstrated that the high-risk group had a worse prognosis (Figures 1C,D). Results from the validation cohorts GSE62254 and GSE183136 were consistent with those from the training cohort (Figures 2A,B,E,F). Univariate Cox regression analysis showed that age (≥65), TNM stage, and risk score were significantly correlated with the prognosis of STAD in GSE62254. Multivariate Cox regression analysis further confirmed the independent prognostic value of the risk score (HR = 2.212, 95%CI: 1.58–3.095, *p* < 0.0001) (Supplementary Table S3). Although the OS difference was not significant at GSE84437 (Figures 2C,D, *p* = 0.095), a significant difference could be obtained if the upper and lower quartiles were compared (Supplementary Figure S2A, *p* = 0.008). Moreover, ROC analysis demonstrated that the AUC of TCGA cohort, GSE62254, GSE84437, and GSE183136 cohorts were 0.62, 0.67, 0.57, and 0.61 (Supplementary Figure S2B, *p* < 0.05).

**TABLE 2:** Univariable and multivariable Cox regression analysis of the SLC-based signature and survival in TCGA dataset.

Variable	Univariable analysis				Multivariable analysis			
	HR	HR.95 L	HR.95H	*p*-value	HR	HR.95 L	HR.95H	*p*-value
Age								
≥65 or <65	1.6688	1.1602	2.4003	0.0058	1.9635	1.3556	2.8440	0.0004
Gender								
Male or Female	1.4033	0.9588	2.0538	0.0812				
Grade								
1 or 2 or 3	1.3304	0.9401	1.8828	0.1071				
TNM Stage								
I or II or III III or IV	1.5492	1.2441	1.9290	0.0001	1.6063	1.1265	2.2904	0.0088
T Stage								
1 or 2 or 3 or 4	1.2549	1.0013	1.5729	0.0487	0.9788	0.7294	1.3135	0.8863
Hematogenous metastasis								
Yes or No	1.8055	0.9707	3.3583	0.0620				
Lymphatic metastasis								
Yes or No	1.7663	1.1469	2.7202	0.0098	0.9379	0.5197	1.6923	0.8313
Risk score								
High or Low	1.5406	1.3085	1.8139	0.0000	1.5623	1.3210	1.8478	0.0000

### Clinical and Molecular Features of Different Risk Groups

**FIGURE 3:**
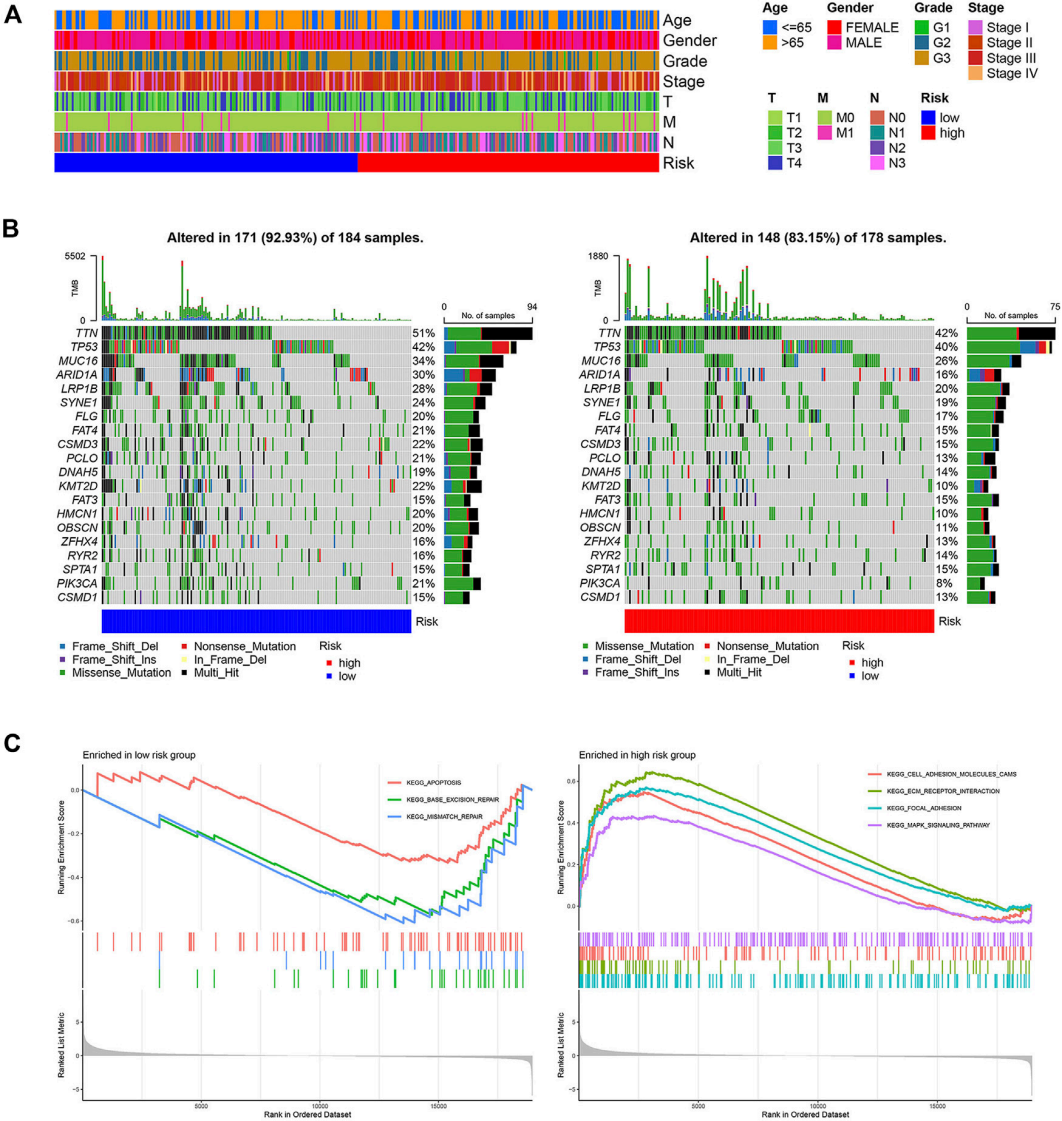
Clinical and molecular characteristics of two risk groups. **(A)** The clinical heatmap of TCGA cohort **(B)** 20 Significantly mutated genes in the mutated STAD samples of two risk groups. The right shows mutation percentage, and the top shows the overall number of mutations. Mutated genes are ordered by mutation rate in TCGA-STAD cohort. **(C)** Gene sets enriched in two groups (*p* < 0.05, FDR<0.25).

The baseline clinical factors were similar for the high- and low-risk groups, as shown in the TCGA clinical heatmap (Figure 3A). However, Lauren’s age and Lauren’s classification were significantly different in the two risk groups in the GSE62254 cohort (Supplementary Figure S3A). Patients with intestinal-type gastric cancer in the low-risk group tended to be older (Supplementary Figures S3B,C). The status of the top 20 commonly mutated genes in the TCGA cohort was checked. The most frequent mutation type was missense mutation, followed by multi-hit mutation. The mutation frequency of those genes in the low-risk group was equal to that of the high-risk group (Figure 3B). GSEA was utilized to clarify the pathway enrichment of two risk groups. Gene sets of the low-risk group were enriched in base excision repair, mismatch repair, and apoptosis pathways. Contrarily, the high-risk groups were enriched for cell adhesion molecules, extracellular matrix (ECM) receptor interaction, focal adhesion, and Mitogen-Activated Protein Kinase (MAPK) signaling pathways, which gave the samples more active proliferation and significant aggressiveness (Figure 3C).

### Kyoto Encyclopedia of Genes and Genomes Analysis of Differential Genes

**FIGURE 4:**
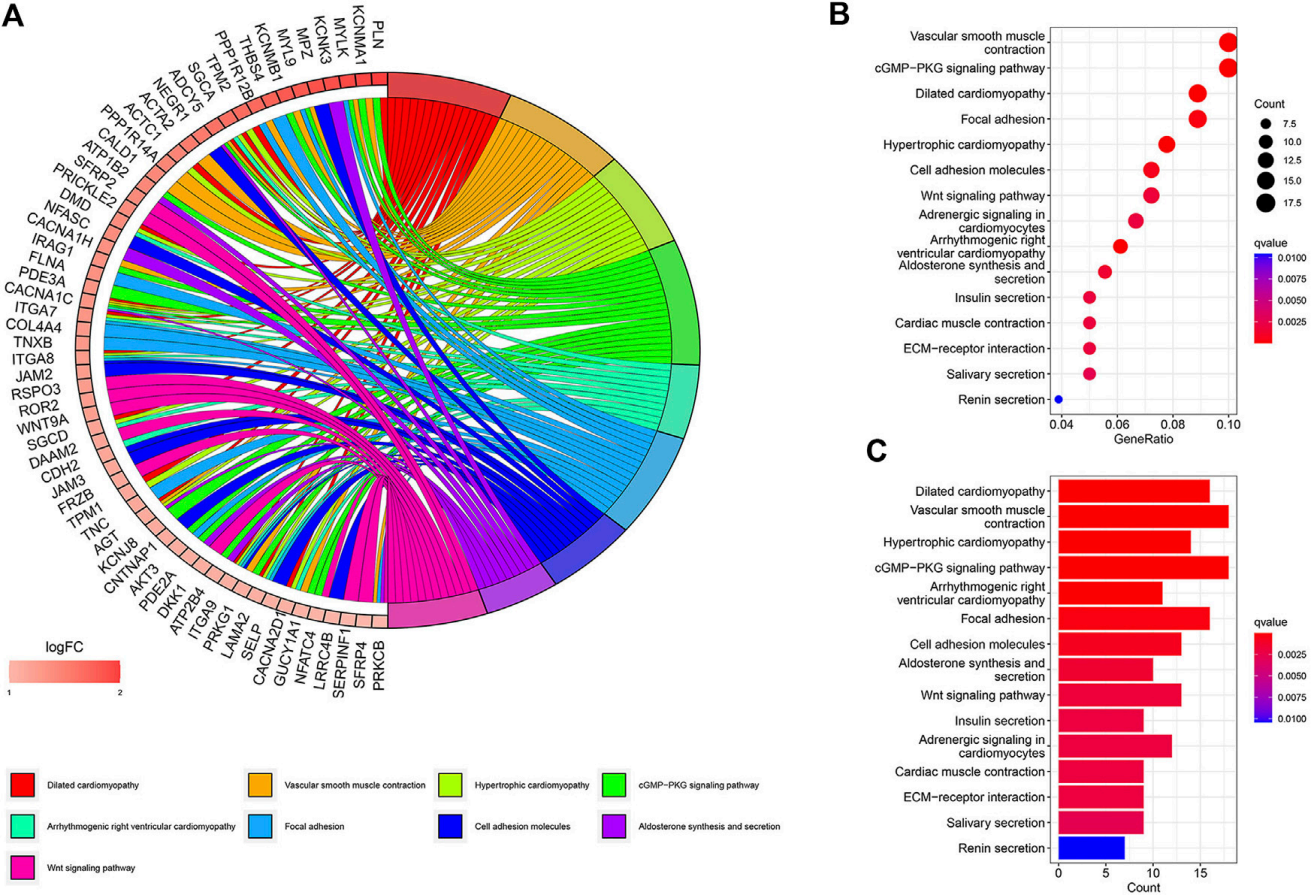
KEGG signaling pathway analysis of differential genes. **(A)** Circos plot. **(B)** Bubble plot. **(C)** Barplot.

A total of 447 differential genes were identified between the two risk groups with log_2_FC > 1, and the high-risk group had an enriched distribution of highly expressed genes. KEGG analysis was performed to compare the difference in signaling pathways. Focal adhesion, cell adhesion molecules, ECM receptor interaction, and Wnt signaling pathways were significantly expressed genes in high-risk groups, suggesting the aggressiveness of tumors (Figure 4).

### Low-Risk Group had More Helper T Cells With Potent Functions

**FIGURE 5:**
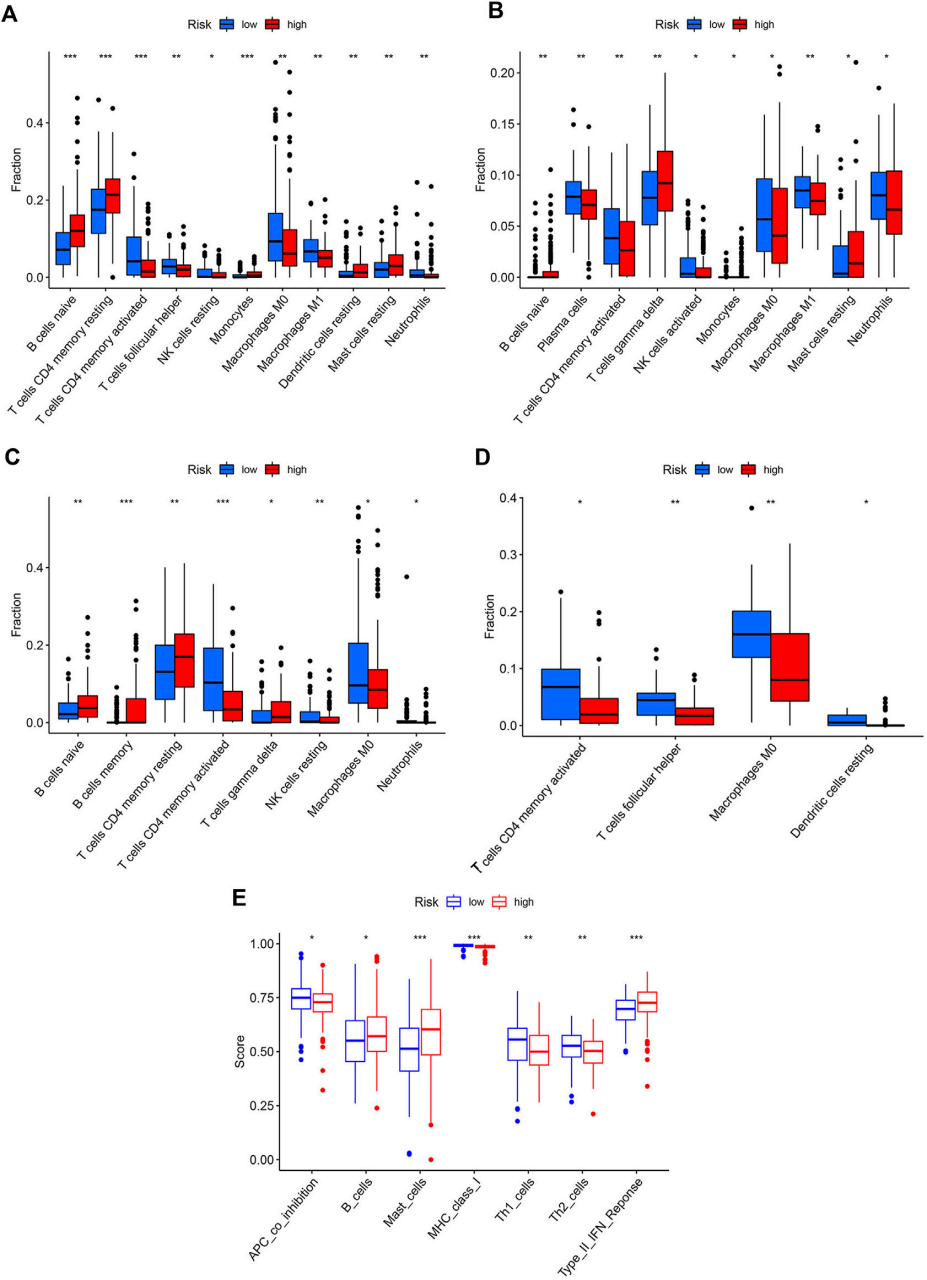
Immune profiles of two risk groups. The immune cell proportion of two risk groups in **(A)** TCGA, **(B)** GSE62254, **(C)** GSE84437, **(D)** GSE18136, **(E)** Immune functions of two risk group based on TCGA cohort.

The correlations between the risk score and the proportion of immune cells were examined by the Wilcoxon test. The high-risk group had higher levels of naïve B cells, resting CD4^+^T memory cells, monocytes, resting DCs, and resting mast cells, while the low-risk group had higher levels of activated CD4^+^ T memory cells, follicular T helper cells, resting NK cells, M0 macrophages, M1 macrophages, and neutrophils (Figure 5A). Combined with the results of three GEO cohorts, activated CD4^+^ T memory cells, M0 macrophages, and neutrophils were significantly higher in the low-risk group than in the high-risk group, while resting CD4^+^T memory cells and naïve B cells showed an opposite trend (Figures 5B–D). The immune functions of two risk groups were assessed based on the TCGA cohort (Figure 5E). The high-risk group had higher functional scores of B cells and mast cells and type II interferon (IFN) response. The low-risk group had more significant APC co-inhibition and stronger class I major histocompatibility complex (MHC) molecules, Th1 cells, and Th2 cells. Similarly, patients with the top 25% of risk scores in three GEO cohorts had significantly higher functional scores of B cells and type II IFN response than patients with the lowest 25% of risk scores (Supplementary Figure S4).

### Low-Risk Group Might Benefit More From ICI Therapy

**FIGURE 6:**
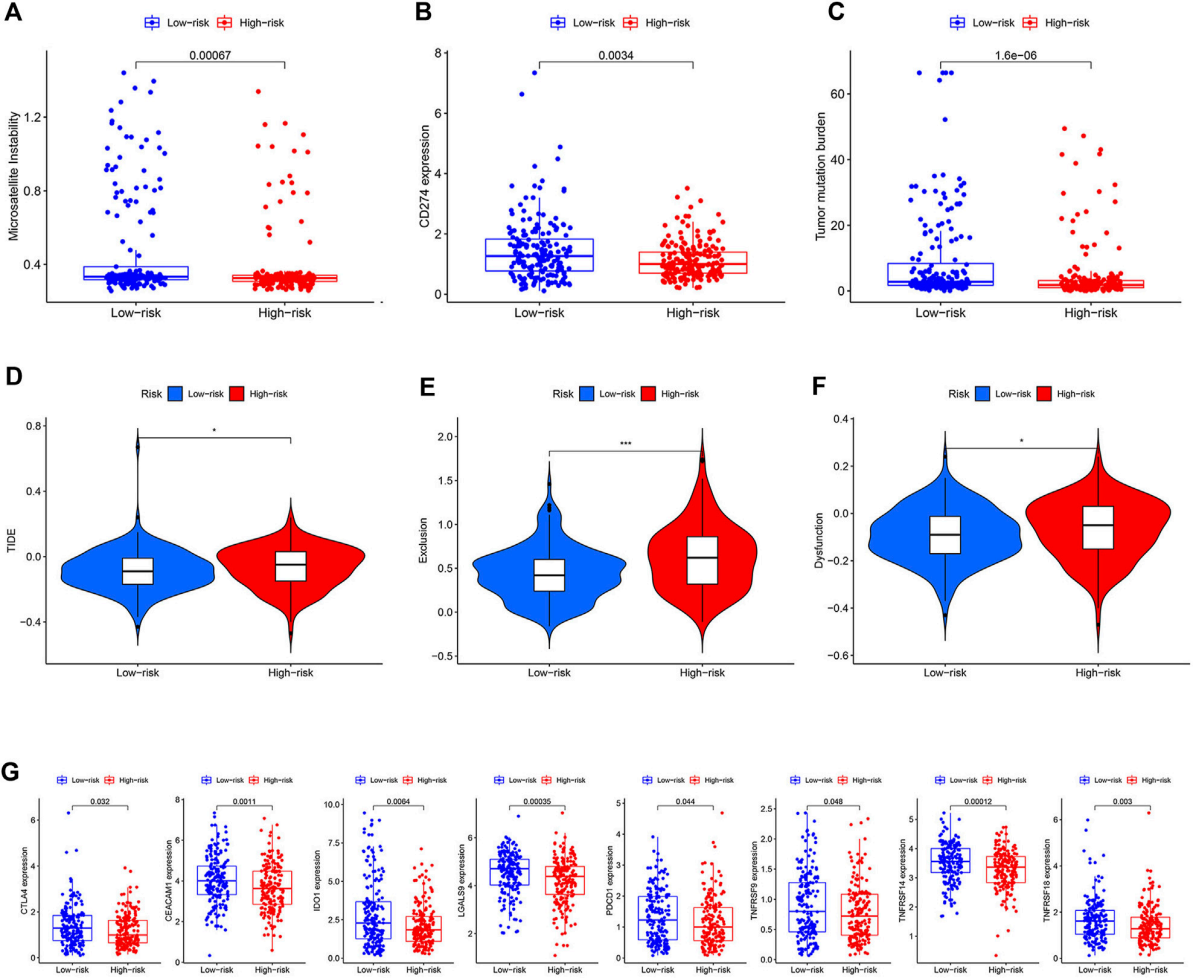
Prediction value of the model. **(A–C)** Difference of MSI, CD274 expression, and TMB between two risk groups. **(D–F)** Difference of TIDE, dysfunction, and exclusion scores between two risk groups. **(G)** Difference of some other co-inhibitor moleculars between two risk groups.

Three widely used biomarkers related to ICI therapy response; microsatellite instability (MSI), CD274 expression, and tumor mutation burden (TMB), were tested in two risk groups in the TCGA cohort. We discovered that patients in the low-risk group had higher MSI, CD274 expression, and TMB, suggesting that ICI therapy might be more beneficial for these patients (Figures 6A–C). Additionally, the TIDE score was introduced into our analysis. The low-risk group, as predicted, had lower TIDE scores, which indicated that patients in this group might acquire more benefits from ICI therapy (Figure 6D). T cell dysfunction and exclusion scores, which together made up the TIDE score, were both lower in the low-risk group (Figures 6E,F). The low-risk group had higher levels of other co-inhibitory molecules such as CTLA4, CEACAM1, IDO1, LGALS9, PDCD1, TNFRSF9, TNFRSF14, and TNFRSF18 (Figure 6G). We then used a cohort (NCT#02589496) comprising 44 GC patients who received anti-PD1 therapy with available clinical information to examine the relationship between ICI therapy response and risk score [26]. Unfortunately, due to the small sample size, we only managed to establish a numerical difference rather than a statistical one (Supplementary Figure S5).

## Discussion

ICI therapy can induce durable response for cancer patients when compared to conventional therapies. However, only one-third of patients respond to ICI in most cancer types [27]. Therefore, more biomarkers are urgently required to evaluate whether patients could benefit from ICI therapy. Henceforth, we constructed a risk score model depending on the expression of specific SLC family members. Four genes such as SLC6A9, SLC7A2, SLC7A3, and SLC25A15, were filtered out to create the model. The risk score had a strong correlation with OS, which the TCGA cohorts and the GEO cohorts both demonstrated. The molecular profile of the high-risk group was helpful for tumor aggressiveness. Additionally, STAD patients with low-risk scores had better T helper functions, higher TMB, and CD274 expression, signifying improved ICI therapy outcomes.

Screened SLC genes play critical roles in cancer development. On the basis of TCGA, SLC6A9 and SLC25A15 were considered protective factors in STAD, while SLC7A2 and SLC7A3 were risk factors. GLYT1, which is primarily a glycine transporter, is encoded by SLC6A9. Kaji et al. evaluated the metabolomic profile of gastric cancer and discovered that patients with low levels of glycine exhibited significantly poor relapse-free survival and 5-year OS [28]. SLC7A2 and SLC7A3 encode CAT2 and CAT3 to deliver arginine, which activates mTORC1 to promote cancer cell growth in response to glutamine starvation [29]. Arginine restriction hinders the migration of gastric cancer cell lines [30]. Moreover, arginine deficiency of T cells prevents the development of central memory T cells and results in CD3zeta chain loss [7, 8]. SLC25A15 encodes a mitochondrial carrier for ornithine and citrulline. Over-expression of SLC25A15 correlates with poor prognosis in bladder urothelial carcinoma and prostate cancer [31, 32]. However, SLC25A15 was identified as a protective factor in this study, which might attribute to some novel signaling pathways and needs further research.

The genetic landscape of high- and low-risk groups was analyzed using GSEA and KEGG. DNA repairing pathways were enriched in the low-risk group. In contrast, ECM receptor interaction, focal adhesion, and Wnt signaling pathways were enriched in the high-risk group. The ECM receptor belonging to the integrin receptors family may connect to the actin cytoskeleton at focal adhesions. ECM binding to integrin activates focal adhesion kinase, which participates in tumorigenesis, angiogenesis, and invasion [25, 33]. Wnt signaling pathway could initiate epithelial to mesenchymal transition for facilitating the aggressiveness of the tumor cells [34]. Furthermore, Wnt signaling might induce T cell exclusion resulting in resistance to ICI therapy [35]. These tumor-promoting pathways might contribute to the poor prognosis of the high-risk group.

In addition, to reflect prognosis, this SLC model has the potential to predict the response to ICI immunotherapy. The low-risk group had a higher proportion of activated CD4^+^ T cells and more efficient Th1 and Th2 cells. Activated CD4^+^ T cells can exhibit significant antitumor functions by releasing a series of cytokines [36]. Th1 cells secrete IFN-γ to facilitate CD8^+^ T cell recruitment and IL-2 to enhance T cell proliferation and granzyme B release [37]. Th2 cells could deprive nutrients of cancer cells by guiding arginase-expressing macrophages to tumor sites consuming arginine [38]. The greater number of efficient T helper cells in the low-risk group could produce a sensitive environment for immunotherapy. On the other hand, the high-risk group had stronger B cells and mast cells and more significant IFN-γ response. Although IFN-γ shows anti-tumor effects, it is reported that sustained IFN- γ expression could induce chronic inflammation to promote immune escape [39]. We hypothesized that the increased functions of B cells, mast cells, and IFN-γ response in the high-risk group might be harmful for ICI treatment partly *via* constant inflammatory stimulations. Their exact roles in the immune microenvironment needs more experimental investigation.

In our study, the low-risk group had significantly higher TMB and PD-L1 expression. According to the KEYNOTE-059 trial, patients with PD-L1 expression had higher objective response rate and longer median response duration [40]. TMB can represent tumor neoantigen, and in several clinical trials, higher TMB was found to be correlated with better ICI therapy response [41]. These findings suggested that low-risk patients might have better ICI therapy response. The low-risk group also presented lower TIDE scores, representing the extent of T-cell dysfunction in cytotoxic T lymphocyte (CTL)-high tumors and T-cell exclusion in CTL-low tumors. TIDE score was positively associated with an immunosuppressive microinvironment [24]. As a result, low-risk patients might benefit more from immunotherapy.

In conclusion, this SLC family-based model is a promising prognostic biomarker and might be a reliable predictor for ICI therapy response, improving the clinical management of STAD patients.

## Data Availability

The original contributions presented in the study are included in the article/Supplementary Material, further inquiries can be directed to the corresponding author.
