# Supplementary figures and images for: Comprehensive Molecular Analyses of an SLC Family-Based Model in Stomach Adenocarcinoma
Source: Pathol Oncol Res. 2022 Oct 13;28:1610610. doi: 10.3389/pore.2022.1610610 (PMC9606230; doi:10.3389/pore.2022.1610610)

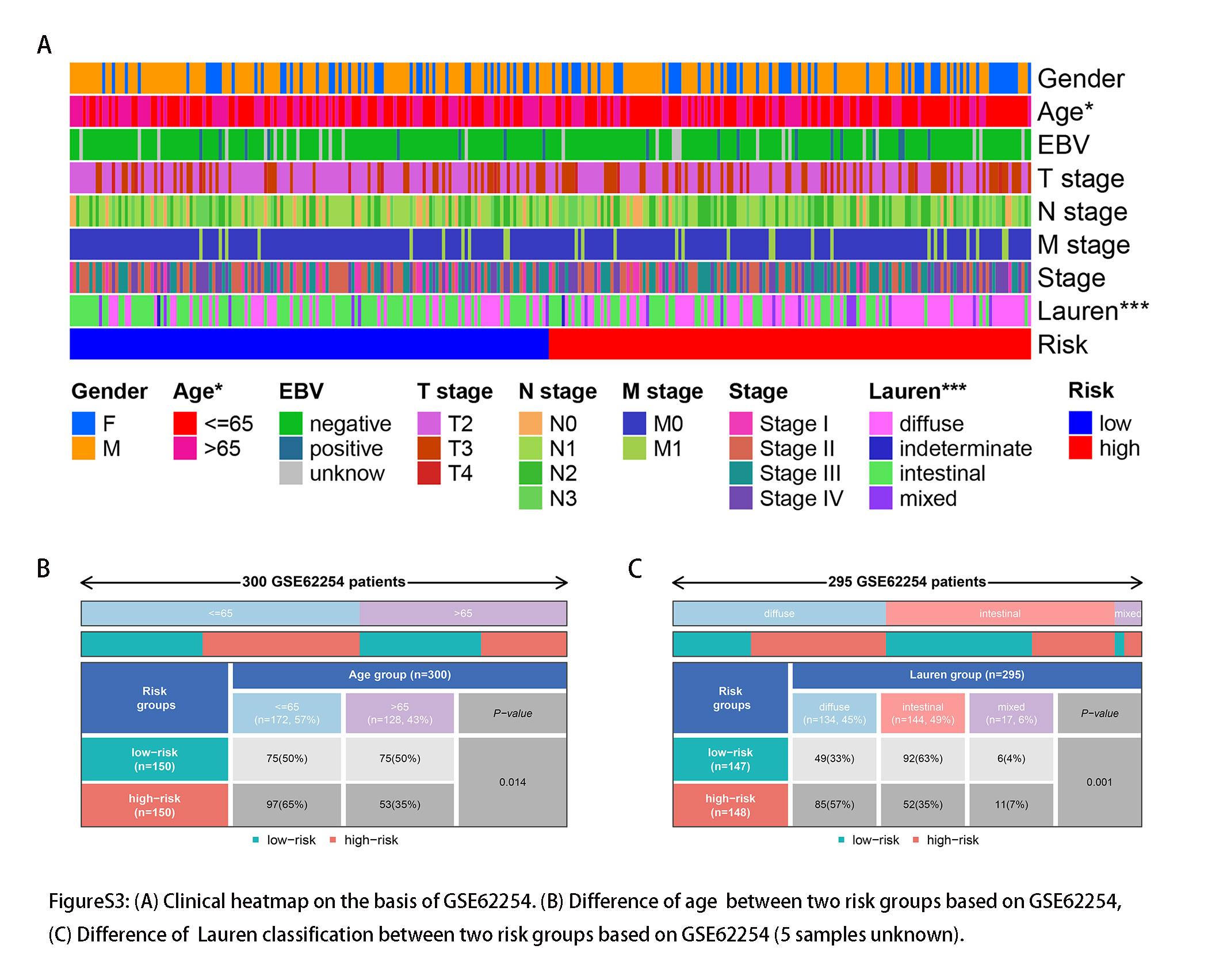

Supplement: Supplementary file 1 [file Image3.JPEG]

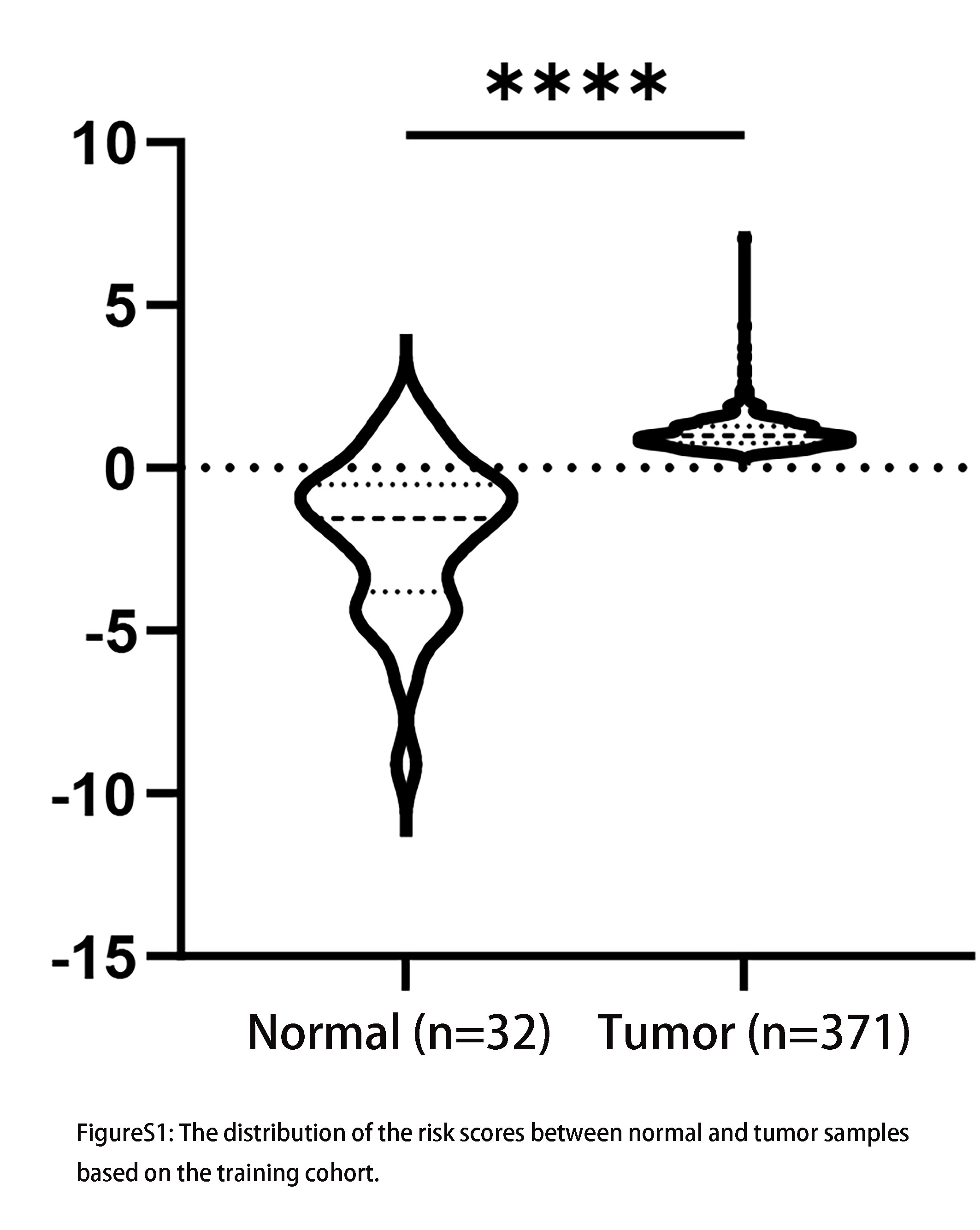

Supplement: Supplementary file 3 [file Image1.JPEG]

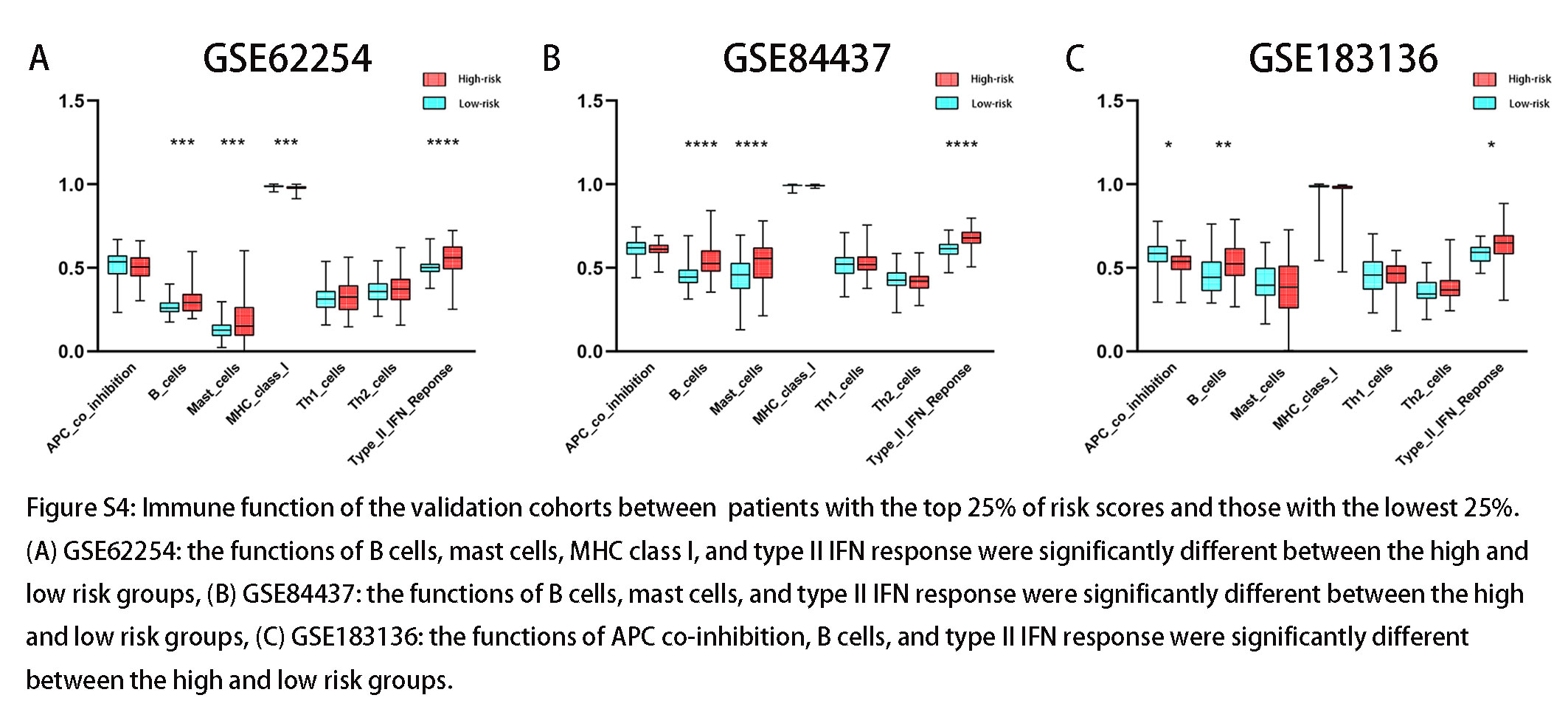

Supplement: Supplementary file 4 [file Image4.JPEG]

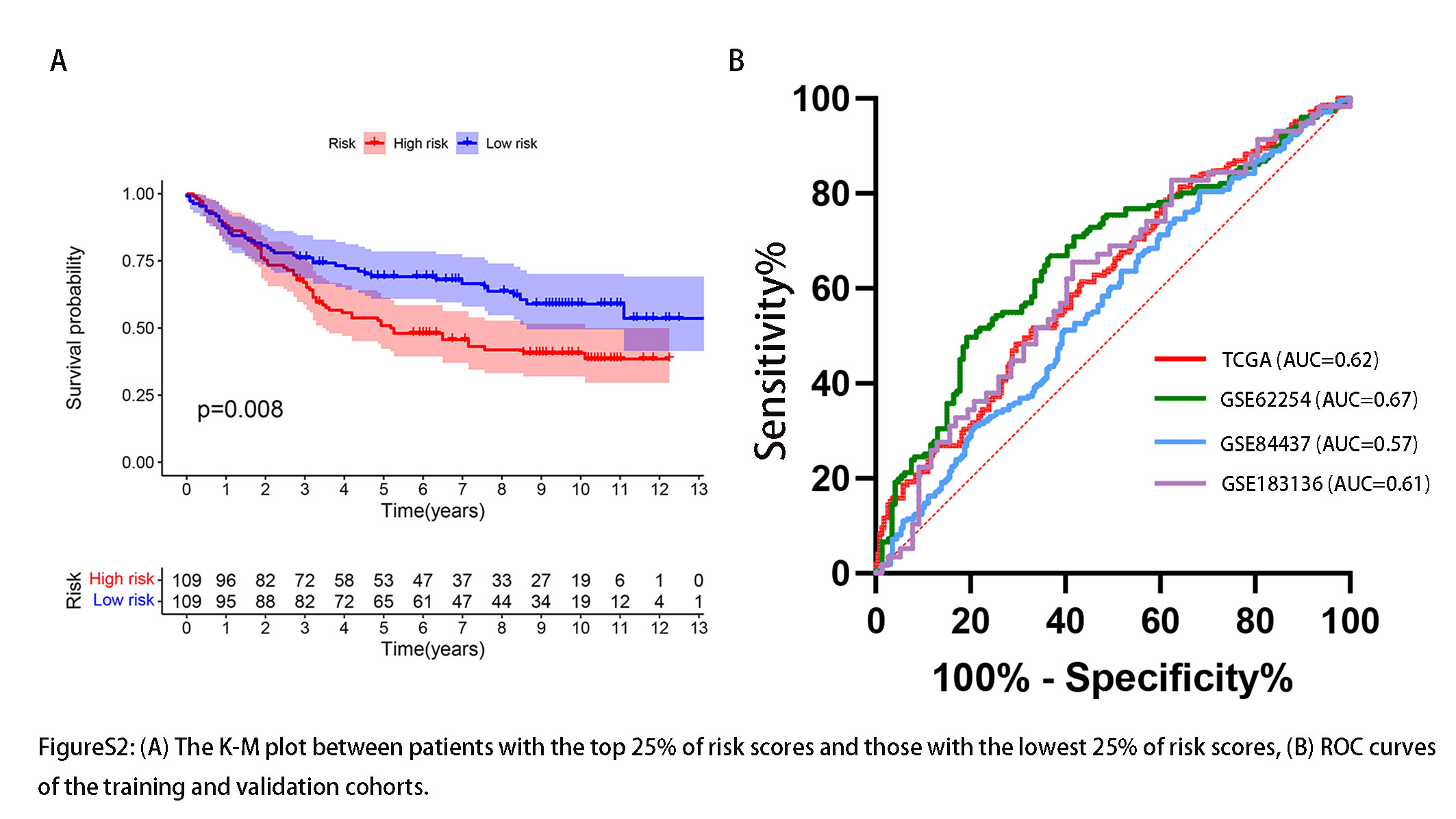

Supplement: Supplementary file 5 [file Image2.JPEG]

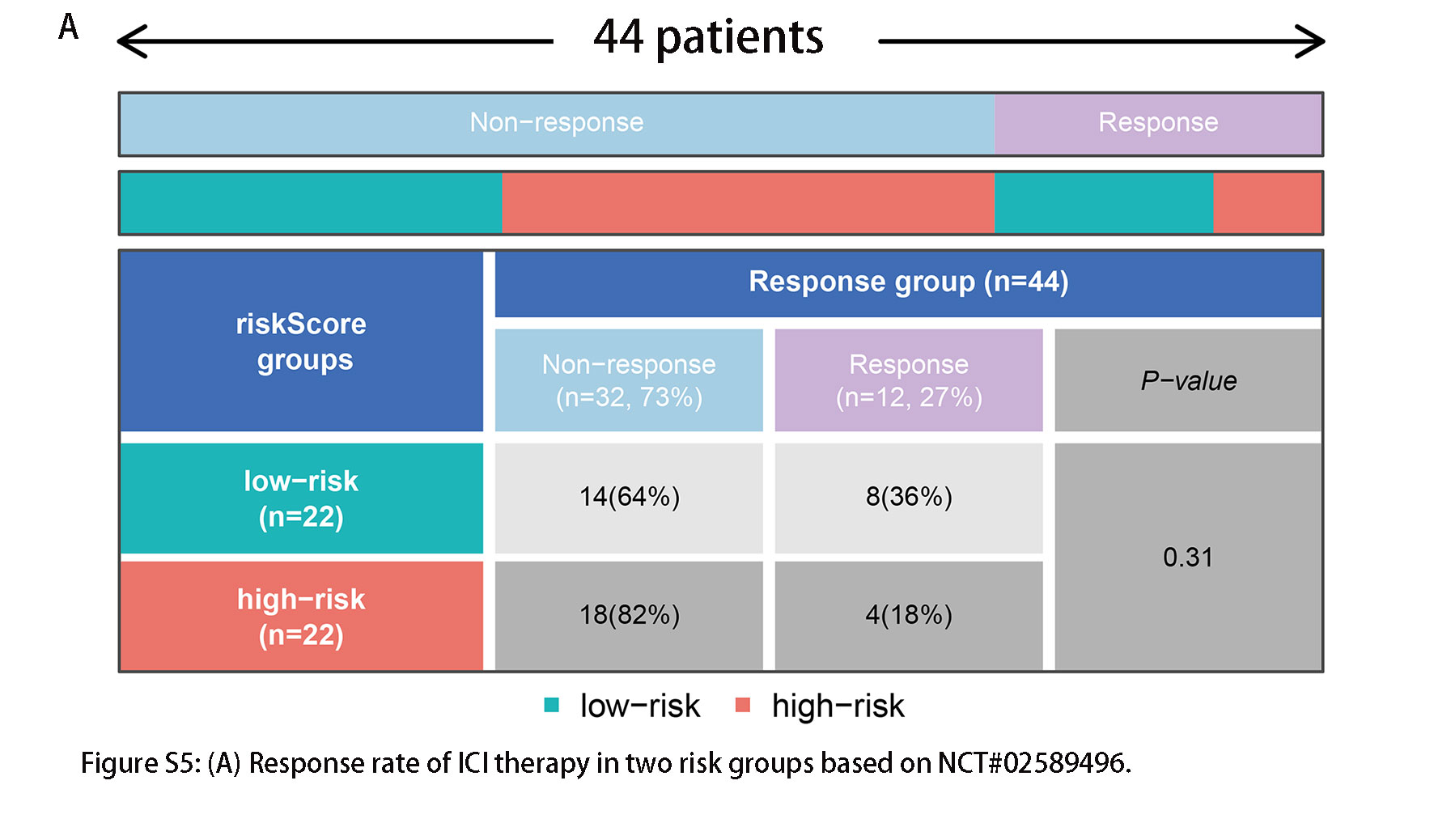

Supplement: Supplementary file 6 [file Image5.JPEG]
