# Supplementary material for: Comprehensive Molecular Analyses of an SLC Family-Based Model in Stomach Adenocarcinoma
Source: Pathol Oncol Res. 2022 Oct 13;28:1610610. doi: 10.3389/pore.2022.1610610 (PMC9606230; doi:10.3389/pore.2022.1610610)
Supplement: Supplementary file 2 [file Table1.DOCX]

**TableS1**

Clinical information of TCGA and GEO cohorts

| TCGA cohort | | | | | | | |
| --- | --- | --- | --- | --- | --- | --- | --- |
| Id | Age | Gender | Grade | Stage | T | M | N |
| TCGA-VQ-A8PX | <=65 | MALE | G2 | Stage I | T1 | M0 | N0 |
| TCGA-CG-4443 | >65 | MALE | G2 | Stage I | T1 | M0 | N0 |
| TCGA-CG-5726 | >65 | MALE | G2 | Stage I | T1 | M0 | N0 |
| TCGA-HF-7134 | unknown | MALE | G2 | Stage I | T1 | M0 | N0 |
| TCGA-HU-8244 | >65 | FEMALE | G1 | Stage I | T1 | M0 | N0 |
| TCGA-HU-A4G9 | >65 | FEMALE | G2 | Stage I | T1 | M0 | N0 |
| TCGA-IN-A6RI | <=65 | MALE | G2 | Stage I | T1 | M0 | N0 |
| TCGA-BR-8486 | >65 | FEMALE | G2 | Stage I | T1 | M0 | N0 |
| TCGA-IN-AB1V | <=65 | MALE | G2 | Stage I | T1 | M0 | N0 |
| TCGA-IN-A6RO | >65 | MALE | G2 | Stage I | T1 | M0 | N0 |
| TCGA-HU-A4GH | >65 | MALE | G2 | Stage I | T1 | M0 | N0 |
| TCGA-HU-8610 | >65 | MALE | G2 | Stage I | T1 | M0 | N0 |
| TCGA-IN-A6RS | >65 | MALE | G2 | Stage I | T1 | M0 | N0 |
| TCGA-BR-7703 | >65 | MALE | G2 | Stage I | T1 | M0 | N0 |
| TCGA-IN-A6RJ | <=65 | MALE | G3 | Stage I | T1 | M0 | N0 |
| TCGA-HU-A4G6 | >65 | MALE | G3 | Stage I | T1 | M0 | N0 |
| TCGA-F1-6875 | >65 | MALE | G2 | Stage I | T2 | M0 | N0 |
| TCGA-KB-A93G | >65 | MALE | G1 | Stage I | T2 | M0 | N0 |
| TCGA-BR-8679 | <=65 | FEMALE | G2 | Stage I | T2 | M0 | N0 |
| TCGA-BR-A4PE | >65 | FEMALE | G2 | Stage I | T2 | M0 | N0 |
| TCGA-D7-6528 | >65 | FEMALE | G2 | Stage I | T2 | M0 | N0 |
| TCGA-BR-6710 | <=65 | MALE | G2 | Stage I | T2 | M0 | N0 |
| TCGA-VQ-A8DV | <=65 | MALE | G2 | Stage I | T2 | M0 | N0 |
| TCGA-CG-4436 | <=65 | MALE | G2 | Stage I | T2 | M0 | N0 |
| TCGA-CD-A4MJ | <=65 | MALE | G2 | Stage I | T2 | M0 | N0 |
| TCGA-D7-8578 | >65 | MALE | G2 | Stage I | T2 | M0 | N0 |
| TCGA-BR-8678 | >65 | MALE | G2 | Stage I | T2 | M0 | N0 |
| TCGA-D7-6822 | >65 | MALE | G2 | Stage I | T2 | M0 | N0 |
| TCGA-RD-A8N2 | <=65 | FEMALE | G3 | Stage I | T2 | M0 | N0 |
| TCGA-RD-A7BW | >65 | FEMALE | G3 | Stage I | T2 | M0 | N0 |
| TCGA-BR-7707 | >65 | FEMALE | G3 | Stage I | T2 | M0 | N0 |
| TCGA-BR-8363 | >65 | FEMALE | G3 | Stage I | T2 | M0 | N0 |
| TCGA-D7-A6F0 | >65 | FEMALE | G3 | Stage I | T2 | M0 | N0 |
| TCGA-BR-8368 | >65 | FEMALE | G3 | Stage I | T2 | M0 | N0 |
| TCGA-CG-5728 | >65 | FEMALE | G3 | Stage I | T2 | M0 | N0 |
| TCGA-D7-6522 | <=65 | MALE | G3 | Stage I | T2 | M0 | N0 |
| TCGA-D7-A6F2 | <=65 | MALE | G3 | Stage I | T2 | M0 | N0 |
| TCGA-B7-5818 | <=65 | MALE | G3 | Stage I | T2 | M0 | N0 |
| TCGA-CG-5720 | >65 | MALE | G3 | Stage I | T2 | M0 | N0 |
| TCGA-CG-5725 | >65 | MALE | G3 | Stage I | T2 | M0 | N0 |
| TCGA-F1-6874 | >65 | MALE | G3 | Stage I | T2 | M0 | N0 |
| TCGA-RD-A7C1 | >65 | MALE | G3 | Stage I | T2 | M0 | N0 |
| TCGA-CG-4442 | >65 | MALE | G3 | Stage I | T2 | M0 | N0 |
| TCGA-SW-A7EA | <=65 | FEMALE | unknown | Stage I | T2 | M0 | N0 |
| TCGA-HU-A4H5 | >65 | MALE | unknown | Stage I | T2 | M0 | N0 |
| TCGA-CG-4466 | >65 | FEMALE | G2 | Stage I | T2 | M0 | N0 |
| TCGA-BR-4267 | <=65 | MALE | G2 | Stage I | T2 | M0 | N0 |
| TCGA-CG-4304 | >65 | MALE | G2 | Stage I | T2 | M0 | N0 |
| TCGA-CG-4477 | <=65 | FEMALE | G3 | Stage I | T2 | M0 | N0 |
| TCGA-FP-8209 | <=65 | MALE | G3 | Stage I | T2 | M0 | N0 |
| TCGA-VQ-A92D | >65 | MALE | G2 | Stage I | T2 | M0 | N0 |
| TCGA-CD-5800 | <=65 | FEMALE | G1 | Stage II | T3 | M0 | N0 |
| TCGA-VQ-A8PB | <=65 | FEMALE | G2 | Stage II | T3 | M0 | N0 |
| TCGA-CD-8532 | <=65 | MALE | G2 | Stage II | T3 | M0 | N0 |
| TCGA-CD-5802 | <=65 | MALE | G2 | Stage II | T3 | M0 | N0 |
| TCGA-VQ-A924 | >65 | MALE | G2 | Stage II | T3 | M0 | N0 |
| TCGA-CD-5798 | >65 | MALE | G2 | Stage II | T3 | M0 | N0 |
| TCGA-VQ-A8PZ | <=65 | FEMALE | G3 | Stage II | T3 | M0 | N0 |
| TCGA-CD-8524 | <=65 | FEMALE | G3 | Stage II | T3 | M0 | N0 |
| TCGA-BR-7704 | >65 | FEMALE | G3 | Stage II | T3 | M0 | N0 |
| TCGA-CD-5803 | >65 | FEMALE | G3 | Stage II | T3 | M0 | N0 |
| TCGA-CD-8534 | <=65 | MALE | G3 | Stage II | T3 | M0 | N0 |
| TCGA-CD-8533 | <=65 | MALE | G3 | Stage II | T3 | M0 | N0 |
| TCGA-BR-8286 | <=65 | MALE | G3 | Stage II | T3 | M0 | N0 |
| TCGA-CD-8530 | <=65 | MALE | G3 | Stage II | T3 | M0 | N0 |
| TCGA-CD-5813 | <=65 | MALE | G3 | Stage II | T3 | M0 | N0 |
| TCGA-CG-5727 | >65 | MALE | G3 | Stage II | T3 | M0 | N0 |
| TCGA-CD-8536 | >65 | MALE | G3 | Stage II | T3 | M0 | N0 |
| TCGA-BR-7197 | >65 | MALE | unknown | Stage II | T3 | M0 | N0 |
| TCGA-VQ-A8DL | >65 | FEMALE | G2 | Stage II | T3 | M0 | N0 |
| TCGA-BR-A4J6 | >65 | FEMALE | G2 | Stage II | T3 | M0 | N0 |
| TCGA-HU-8604 | >65 | FEMALE | G2 | Stage II | T3 | M0 | N0 |
| TCGA-CD-A4MH | >65 | FEMALE | G2 | Stage II | T3 | M0 | N0 |
| TCGA-CD-A48A | <=65 | MALE | G2 | Stage II | T3 | M0 | N0 |
| TCGA-BR-7715 | <=65 | MALE | G2 | Stage II | T3 | M0 | N0 |
| TCGA-BR-8360 | >65 | MALE | G2 | Stage II | T3 | M0 | N0 |
| TCGA-CD-A486 | >65 | MALE | G2 | Stage II | T3 | M0 | N0 |
| TCGA-VQ-AA6G | >65 | MALE | G2 | Stage II | T3 | M0 | N0 |
| TCGA-F1-A72C | >65 | MALE | G2 | Stage II | T3 | M0 | N0 |
| TCGA-HU-A4GF | >65 | MALE | G2 | Stage II | T3 | M0 | N0 |
| TCGA-BR-6801 | >65 | MALE | G2 | Stage II | T3 | M0 | N0 |
| TCGA-BR-A44T | <=65 | FEMALE | G3 | Stage II | T3 | M0 | N0 |
| TCGA-BR-6803 | <=65 | FEMALE | G3 | Stage II | T3 | M0 | N0 |
| TCGA-BR-6852 | <=65 | FEMALE | G3 | Stage II | T3 | M0 | N0 |
| TCGA-BR-8487 | <=65 | FEMALE | G3 | Stage II | T3 | M0 | N0 |
| TCGA-BR-6566 | <=65 | FEMALE | G3 | Stage II | T3 | M0 | N0 |
| TCGA-BR-A4QI | >65 | FEMALE | G3 | Stage II | T3 | M0 | N0 |
| TCGA-BR-8365 | >65 | FEMALE | G3 | Stage II | T3 | M0 | N0 |
| TCGA-HU-A4GT | >65 | FEMALE | G3 | Stage II | T3 | M0 | N0 |
| TCGA-IN-AB1X | >65 | FEMALE | G3 | Stage II | T3 | M0 | N0 |
| TCGA-BR-6452 | >65 | FEMALE | G3 | Stage II | T3 | M0 | N0 |
| TCGA-BR-8366 | >65 | FEMALE | G3 | Stage II | T3 | M0 | N0 |
| TCGA-BR-A4J9 | <=65 | MALE | G3 | Stage II | T3 | M0 | N0 |
| TCGA-FP-A4BE | <=65 | MALE | G3 | Stage II | T3 | M0 | N0 |
| TCGA-D7-8573 | <=65 | MALE | G3 | Stage II | T3 | M0 | N0 |
| TCGA-BR-6454 | <=65 | MALE | G3 | Stage II | T3 | M0 | N0 |
| TCGA-CD-A489 | <=65 | MALE | G3 | Stage II | T3 | M0 | N0 |
| TCGA-MX-A663 | >65 | MALE | G3 | Stage II | T3 | M0 | N0 |
| TCGA-VQ-A8P5 | >65 | MALE | G3 | Stage II | T3 | M0 | N0 |
| TCGA-BR-6457 | >65 | MALE | G3 | Stage II | T3 | M0 | N0 |
| TCGA-HU-8245 | >65 | MALE | G3 | Stage II | T3 | M0 | N0 |
| TCGA-BR-6707 | >65 | MALE | G3 | Stage II | T3 | M0 | N0 |
| TCGA-CD-A4MG | >65 | MALE | G3 | Stage II | T3 | M0 | N0 |
| TCGA-VQ-A8E3 | >65 | MALE | G3 | Stage II | T3 | M0 | N0 |
| TCGA-CD-8528 | <=65 | FEMALE | G3 | Stage III | T4 | M0 | N0 |
| TCGA-BR-4361 | >65 | FEMALE | G3 | Stage III | T4 | M0 | N0 |
| TCGA-BR-8081 | >65 | FEMALE | G2 | Stage II | T4 | M0 | N0 |
| TCGA-VQ-A8P8 | >65 | FEMALE | G2 | Stage II | T4 | M0 | N0 |
| TCGA-BR-6565 | >65 | MALE | G2 | Stage II | T4 | M0 | N0 |
| TCGA-B7-5816 | <=65 | FEMALE | G3 | Stage II | T4 | M0 | N0 |
| TCGA-BR-8588 | <=65 | FEMALE | G3 | Stage II | T4 | M0 | N0 |
| TCGA-HU-8602 | <=65 | FEMALE | G3 | Stage II | T4 | M0 | N0 |
| TCGA-BR-8295 | <=65 | FEMALE | G3 | Stage II | T4 | M0 | N0 |
| TCGA-BR-8078 | >65 | FEMALE | G3 | Stage II | T4 | M0 | N0 |
| TCGA-BR-A4PD | >65 | FEMALE | G3 | Stage II | T4 | M0 | N0 |
| TCGA-BR-8682 | <=65 | MALE | G3 | Stage II | T4 | M0 | N0 |
| TCGA-BR-A4IY | <=65 | MALE | G3 | Stage II | T4 | M0 | N0 |
| TCGA-BR-A4J2 | >65 | MALE | G3 | Stage II | T4 | M0 | N0 |
| TCGA-VQ-A94U | >65 | MALE | G3 | Stage II | T4 | M0 | N0 |
| TCGA-BR-7851 | >65 | MALE | G3 | Stage II | T4 | M0 | N0 |
| TCGA-VQ-A8PO | >65 | MALE | G3 | Stage II | T4 | M0 | N0 |
| TCGA-VQ-A91E | >65 | FEMALE | G3 | Stage III | T4 | M0 | N0 |
| TCGA-BR-7958 | <=65 | MALE | G3 | Stage III | T4 | M0 | N0 |
| TCGA-VQ-A8PT | <=65 | MALE | G3 | Stage III | T4 | M0 | N0 |
| TCGA-CG-5719 | <=65 | FEMALE | G1 | Stage IV | T4 | M1 | N0 |
| TCGA-CD-8529 | <=65 | MALE | G2 | Stage IV | T4 | M1 | N0 |
| TCGA-ZA-A8F6 | >65 | MALE | G2 | Stage I | T2 | unknown | N0 |
| TCGA-FP-7735 | >65 | MALE | G2 | Stage I | T2 | unknown | N0 |
| TCGA-HF-7131 | unknown | FEMALE | G2 | Stage I | T2 | unknown | N0 |
| TCGA-3M-AB46 | >65 | MALE | G2 | Stage I | T2 | unknown | N0 |
| TCGA-HJ-7597 | >65 | FEMALE | G3 | Stage I | T2 | unknown | N0 |
| TCGA-FP-8099 | >65 | MALE | G2 | Stage II | T3 | unknown | N0 |
| TCGA-KB-A6F7 | <=65 | FEMALE | G3 | Stage I | T1 | M0 | N1 |
| TCGA-HU-A4H8 | >65 | MALE | G2 | Stage I | T1 | M0 | N1 |
| TCGA-FP-A9TM | >65 | MALE | G2 | unknown | T1 | M0 | N1 |
| TCGA-CD-8527 | >65 | FEMALE | G2 | Stage II | T2 | M0 | N1 |
| TCGA-D7-6527 | <=65 | MALE | G2 | Stage II | T2 | M0 | N1 |
| TCGA-CG-4449 | >65 | MALE | G2 | Stage II | T2 | M0 | N1 |
| TCGA-CG-4437 | >65 | MALE | G2 | Stage II | T2 | M0 | N1 |
| TCGA-RD-A8N9 | <=65 | FEMALE | G3 | Stage II | T2 | M0 | N1 |
| TCGA-CD-5799 | <=65 | MALE | G3 | Stage II | T2 | M0 | N1 |
| TCGA-D7-6524 | <=65 | MALE | G3 | Stage II | T2 | M0 | N1 |
| TCGA-CG-5723 | >65 | MALE | G3 | Stage II | T2 | M0 | N1 |
| TCGA-HU-A4GP | <=65 | FEMALE | G2 | Stage II | T2 | M0 | N1 |
| TCGA-HU-A4GN | <=65 | MALE | G2 | Stage II | T2 | M0 | N1 |
| TCGA-BR-6453 | <=65 | MALE | G3 | Stage II | T2 | M0 | N1 |
| TCGA-D7-6820 | <=65 | MALE | G2 | Stage II | T2 | M0 | N1 |
| TCGA-IN-8462 | >65 | MALE | G2 | Stage II | T2 | M0 | N1 |
| TCGA-IN-A6RL | >65 | MALE | G2 | unknown | T2 | M0 | N1 |
| TCGA-D7-6519 | <=65 | FEMALE | G3 | unknown | T2 | M0 | N1 |
| TCGA-BR-4279 | <=65 | MALE | G3 | Stage II | T2 | M0 | N1 |
| TCGA-CG-4305 | >65 | MALE | G3 | Stage II | T2 | M0 | N1 |
| TCGA-CG-5718 | >65 | FEMALE | G2 | Stage II | T2 | M0 | N1 |
| TCGA-HF-7132 | unknown | MALE | G2 | Stage II | T2 | M0 | N1 |
| TCGA-CG-5717 | <=65 | MALE | G3 | Stage II | T2 | M0 | N1 |
| TCGA-CG-4455 | >65 | MALE | G3 | Stage II | T2 | M0 | N1 |
| TCGA-KB-A93J | >65 | MALE | G3 | Stage II | T2 | M0 | N1 |
| TCGA-BR-4280 | >65 | FEMALE | G2 | Stage III | T2 | M0 | N1 |
| TCGA-KB-A93H | >65 | FEMALE | G1 | Stage II | T3 | M0 | N1 |
| TCGA-BR-7716 | <=65 | FEMALE | G2 | Stage II | T3 | M0 | N1 |
| TCGA-BR-6456 | >65 | FEMALE | G2 | Stage II | T3 | M0 | N1 |
| TCGA-VQ-AA6F | <=65 | MALE | G2 | Stage II | T3 | M0 | N1 |
| TCGA-FP-7829 | >65 | MALE | G2 | Stage II | T3 | M0 | N1 |
| TCGA-BR-7901 | >65 | MALE | G2 | Stage II | T3 | M0 | N1 |
| TCGA-BR-6458 | <=65 | FEMALE | G3 | Stage II | T3 | M0 | N1 |
| TCGA-D7-A4YV | >65 | FEMALE | G3 | Stage II | T3 | M0 | N1 |
| TCGA-HU-A4G8 | >65 | FEMALE | G3 | Stage II | T3 | M0 | N1 |
| TCGA-IN-A7NT | >65 | FEMALE | G3 | Stage II | T3 | M0 | N1 |
| TCGA-CD-A48C | >65 | FEMALE | G3 | Stage II | T3 | M0 | N1 |
| TCGA-HU-A4G2 | <=65 | MALE | G3 | Stage II | T3 | M0 | N1 |
| TCGA-IN-7806 | <=65 | MALE | G3 | Stage II | T3 | M0 | N1 |
| TCGA-BR-8381 | <=65 | MALE | G3 | Stage II | T3 | M0 | N1 |
| TCGA-CD-A487 | <=65 | MALE | G3 | Stage II | T3 | M0 | N1 |
| TCGA-HU-A4GD | <=65 | MALE | G3 | Stage II | T3 | M0 | N1 |
| TCGA-D7-A747 | <=65 | MALE | G3 | Stage II | T3 | M0 | N1 |
| TCGA-BR-6455 | <=65 | MALE | G3 | Stage II | T3 | M0 | N1 |
| TCGA-BR-6563 | <=65 | MALE | G3 | Stage II | T3 | M0 | N1 |
| TCGA-BR-7722 | <=65 | MALE | G3 | Stage II | T3 | M0 | N1 |
| TCGA-D7-A4YX | <=65 | MALE | G3 | Stage II | T3 | M0 | N1 |
| TCGA-HU-A4GU | >65 | MALE | G3 | Stage II | T3 | M0 | N1 |
| TCGA-CG-4475 | >65 | MALE | G3 | Stage II | T3 | M0 | N1 |
| TCGA-BR-8291 | <=65 | MALE | unknown | Stage II | T3 | M0 | N1 |
| TCGA-CD-A4MI | <=65 | MALE | G1 | Stage III | T3 | M0 | N1 |
| TCGA-VQ-A91Z | >65 | FEMALE | G2 | Stage III | T3 | M0 | N1 |
| TCGA-VQ-A91W | <=65 | MALE | G2 | Stage III | T3 | M0 | N1 |
| TCGA-VQ-A91K | >65 | MALE | G2 | Stage III | T3 | M0 | N1 |
| TCGA-VQ-A8PS | >65 | MALE | G2 | Stage III | T3 | M0 | N1 |
| TCGA-RD-A8N5 | >65 | MALE | G2 | Stage III | T3 | M0 | N1 |
| TCGA-VQ-A91U | >65 | MALE | G2 | Stage III | T3 | M0 | N1 |
| TCGA-HF-7136 | unknown | MALE | G2 | Stage III | T3 | M0 | N1 |
| TCGA-BR-4183 | <=65 | FEMALE | G3 | Stage III | T3 | M0 | N1 |
| TCGA-RD-A8N4 | <=65 | FEMALE | G3 | Stage III | T3 | M0 | N1 |
| TCGA-CD-8531 | >65 | FEMALE | G3 | Stage III | T3 | M0 | N1 |
| TCGA-CD-8526 | >65 | FEMALE | G3 | Stage III | T3 | M0 | N1 |
| TCGA-BR-4255 | >65 | FEMALE | G3 | Stage III | T3 | M0 | N1 |
| TCGA-BR-4253 | >65 | FEMALE | G3 | Stage III | T3 | M0 | N1 |
| TCGA-RD-A8NB | >65 | FEMALE | G3 | Stage III | T3 | M0 | N1 |
| TCGA-CD-8525 | >65 | FEMALE | G3 | Stage III | T3 | M0 | N1 |
| TCGA-RD-A7BS | <=65 | MALE | G3 | Stage III | T3 | M0 | N1 |
| TCGA-FP-8210 | <=65 | MALE | G3 | Stage III | T3 | M0 | N1 |
| TCGA-CD-8535 | <=65 | MALE | G3 | Stage III | T3 | M0 | N1 |
| TCGA-BR-6706 | <=65 | MALE | G3 | Stage III | T3 | M0 | N1 |
| TCGA-VQ-A8PC | <=65 | MALE | G3 | Stage III | T3 | M0 | N1 |
| TCGA-CG-5734 | >65 | MALE | G3 | Stage III | T3 | M0 | N1 |
| TCGA-CD-5801 | >65 | MALE | G3 | Stage III | T3 | M0 | N1 |
| TCGA-MX-A5UG | >65 | MALE | G3 | Stage III | T3 | M0 | N1 |
| TCGA-VQ-A923 | >65 | MALE | G3 | Stage III | T3 | M0 | N1 |
| TCGA-CD-5804 | >65 | MALE | G2 | unknown | T3 | M0 | N1 |
| TCGA-IN-A6RR | >65 | MALE | G3 | unknown | T3 | M0 | N1 |
| TCGA-BR-8059 | >65 | MALE | G3 | Stage III | T4 | M0 | N1 |
| TCGA-VQ-A8P3 | >65 | MALE | G2 | Stage III | T4 | M0 | N1 |
| TCGA-HU-A4GY | >65 | FEMALE | G3 | Stage III | T4 | M0 | N1 |
| TCGA-BR-7717 | <=65 | MALE | G2 | Stage IV | T4 | M0 | N1 |
| TCGA-VQ-A922 | >65 | MALE | G2 | Stage IV | T4 | M0 | N1 |
| TCGA-VQ-A8PP | >65 | MALE | G2 | Stage IV | T4 | M0 | N1 |
| TCGA-CG-4472 | <=65 | MALE | G3 | Stage IV | T4 | M0 | N1 |
| TCGA-CG-5721 | <=65 | MALE | G3 | Stage IV | T4 | M0 | N1 |
| TCGA-VQ-AA6D | <=65 | FEMALE | G2 | Stage III | T4 | M0 | N1 |
| TCGA-BR-8373 | <=65 | FEMALE | G2 | Stage III | T4 | M0 | N1 |
| TCGA-BR-8484 | <=65 | MALE | G2 | Stage III | T4 | M0 | N1 |
| TCGA-VQ-A8P2 | >65 | MALE | G2 | Stage III | T4 | M0 | N1 |
| TCGA-BR-A4IU | <=65 | FEMALE | G3 | Stage III | T4 | M0 | N1 |
| TCGA-VQ-A8PU | >65 | FEMALE | G3 | Stage III | T4 | M0 | N1 |
| TCGA-BR-A4J5 | <=65 | MALE | G3 | Stage III | T4 | M0 | N1 |
| TCGA-BR-A452 | <=65 | MALE | G3 | Stage III | T4 | M0 | N1 |
| TCGA-BR-A4CQ | <=65 | MALE | G3 | Stage III | T4 | M0 | N1 |
| TCGA-BR-7959 | <=65 | MALE | G3 | Stage III | T4 | M0 | N1 |
| TCGA-BR-A4J1 | <=65 | MALE | G3 | Stage III | T4 | M0 | N1 |
| TCGA-BR-8077 | <=65 | FEMALE | G2 | Stage III | T4 | M0 | N1 |
| TCGA-BR-8592 | <=65 | FEMALE | G3 | Stage III | T4 | M0 | N1 |
| TCGA-BR-8589 | <=65 | MALE | G3 | Stage III | T4 | M0 | N1 |
| TCGA-BR-8686 | >65 | MALE | G3 | Stage III | T4 | M0 | N1 |
| TCGA-VQ-A91S | <=65 | MALE | unknown | Stage III | T4 | M0 | N1 |
| TCGA-BR-4191 | >65 | MALE | G2 | unknown | unknown | M0 | N1 |
| TCGA-BR-4256 | >65 | MALE | G2 | unknown | unknown | M0 | N1 |
| TCGA-BR-4188 | <=65 | FEMALE | G3 | unknown | unknown | M0 | N1 |
| TCGA-BR-4362 | >65 | FEMALE | G3 | unknown | unknown | M0 | N1 |
| TCGA-BR-4367 | >65 | MALE | G3 | unknown | unknown | M0 | N1 |
| TCGA-CG-5730 | >65 | FEMALE | G2 | Stage IV | T2 | M1 | N1 |
| TCGA-R5-A7O7 | <=65 | MALE | G2 | Stage IV | T3 | M1 | N1 |
| TCGA-VQ-A8E7 | <=65 | MALE | G2 | Stage IV | T3 | M1 | N1 |
| TCGA-VQ-A8PQ | <=65 | FEMALE | G2 | Stage IV | T4 | M1 | N1 |
| TCGA-CG-4460 | >65 | FEMALE | G2 | Stage IV | T4 | M1 | N1 |
| TCGA-R5-A7ZI | <=65 | FEMALE | G3 | Stage IV | T4 | M1 | N1 |
| TCGA-CG-4301 | >65 | FEMALE | G3 | Stage IV | T4 | M1 | N1 |
| TCGA-R5-A7ZF | <=65 | FEMALE | G2 | Stage IV | T4 | M1 | N1 |
| TCGA-F1-6177 | >65 | MALE | G2 | Stage I | T1 | unknown | N1 |
| TCGA-FP-8211 | <=65 | MALE | G2 | Stage II | T3 | unknown | N1 |
| TCGA-EQ-8122 | >65 | FEMALE | G3 | Stage II | T3 | unknown | N1 |
| TCGA-BR-4184 | >65 | MALE | G2 | Stage III | T3 | unknown | N1 |
| TCGA-EQ-A4SO | >65 | MALE | G2 | Stage III | T4 | unknown | N1 |
| TCGA-IN-A6RN | >65 | FEMALE | G2 | Stage II | T1 | M0 | N2 |
| TCGA-D7-8579 | >65 | FEMALE | G2 | Stage II | T2 | M0 | N2 |
| TCGA-D7-6815 | >65 | FEMALE | G2 | Stage II | T2 | M0 | N2 |
| TCGA-HU-A4G3 | <=65 | MALE | G2 | Stage II | T2 | M0 | N2 |
| TCGA-D7-8572 | <=65 | MALE | G2 | Stage II | T2 | M0 | N2 |
| TCGA-HU-A4H4 | <=65 | FEMALE | G3 | Stage II | T2 | M0 | N2 |
| TCGA-D7-A4Z0 | <=65 | FEMALE | G3 | Stage II | T2 | M0 | N2 |
| TCGA-D7-A6EV | >65 | FEMALE | G3 | Stage II | T2 | M0 | N2 |
| TCGA-BR-8060 | >65 | FEMALE | G3 | Stage II | T2 | M0 | N2 |
| TCGA-IN-8663 | >65 | MALE | G3 | Stage II | T2 | M0 | N2 |
| TCGA-HU-A4HB | >65 | MALE | G3 | Stage II | T2 | M0 | N2 |
| TCGA-CG-4441 | >65 | MALE | G2 | Stage III | T2 | M0 | N2 |
| TCGA-RD-A8N6 | >65 | FEMALE | G3 | Stage III | T2 | M0 | N2 |
| TCGA-CG-5733 | >65 | FEMALE | G3 | Stage III | T2 | M0 | N2 |
| TCGA-D7-6525 | <=65 | MALE | G3 | Stage III | T2 | M0 | N2 |
| TCGA-D7-6520 | <=65 | MALE | G3 | Stage III | T2 | M0 | N2 |
| TCGA-CG-4444 | >65 | MALE | G3 | Stage III | T2 | M0 | N2 |
| TCGA-D7-6521 | <=65 | MALE | G3 | unknown | T2 | M0 | N2 |
| TCGA-D7-A74A | <=65 | FEMALE | G2 | Stage III | T3 | M0 | N2 |
| TCGA-R5-A7ZE | >65 | FEMALE | G2 | Stage III | T3 | M0 | N2 |
| TCGA-D7-6526 | >65 | FEMALE | G2 | Stage III | T3 | M0 | N2 |
| TCGA-HU-A4H6 | >65 | FEMALE | G2 | Stage III | T3 | M0 | N2 |
| TCGA-VQ-AA69 | <=65 | MALE | G2 | Stage III | T3 | M0 | N2 |
| TCGA-SW-A7EB | <=65 | MALE | G2 | Stage III | T3 | M0 | N2 |
| TCGA-VQ-A91V | <=65 | MALE | G2 | Stage III | T3 | M0 | N2 |
| TCGA-BR-8483 | <=65 | MALE | G2 | Stage III | T3 | M0 | N2 |
| TCGA-VQ-A8DU | <=65 | MALE | G2 | Stage III | T3 | M0 | N2 |
| TCGA-VQ-A925 | >65 | MALE | G2 | Stage III | T3 | M0 | N2 |
| TCGA-HU-A4HD | >65 | MALE | G2 | Stage III | T3 | M0 | N2 |
| TCGA-HU-8249 | >65 | MALE | G2 | Stage III | T3 | M0 | N2 |
| TCGA-BR-6564 | <=65 | FEMALE | G3 | Stage III | T3 | M0 | N2 |
| TCGA-D7-A6EX | >65 | FEMALE | G3 | Stage III | T3 | M0 | N2 |
| TCGA-MX-A5UJ | >65 | FEMALE | G3 | Stage III | T3 | M0 | N2 |
| TCGA-BR-6802 | <=65 | MALE | G3 | Stage III | T3 | M0 | N2 |
| TCGA-BR-A4QM | <=65 | MALE | G3 | Stage III | T3 | M0 | N2 |
| TCGA-D7-A6EZ | >65 | MALE | G3 | Stage III | T3 | M0 | N2 |
| TCGA-FP-A4BF | >65 | MALE | G3 | Stage III | T3 | M0 | N2 |
| TCGA-VQ-A8E0 | >65 | MALE | G3 | Stage III | T3 | M0 | N2 |
| TCGA-KB-A6F5 | >65 | MALE | G3 | Stage III | T3 | M0 | N2 |
| TCGA-D7-5579 | >65 | MALE | G3 | Stage III | T3 | M0 | N2 |
| TCGA-D7-8575 | >65 | MALE | G3 | Stage III | T3 | M0 | N2 |
| TCGA-D7-5578 | >65 | MALE | G3 | Stage III | T3 | M0 | N2 |
| TCGA-VQ-A8PY | <=65 | FEMALE | G2 | Stage III | T3 | M0 | N2 |
| TCGA-VQ-AA6B | <=65 | MALE | G2 | Stage III | T3 | M0 | N2 |
| TCGA-VQ-A8E2 | <=65 | MALE | G2 | Stage III | T3 | M0 | N2 |
| TCGA-VQ-AA64 | >65 | MALE | G2 | Stage III | T3 | M0 | N2 |
| TCGA-CG-4300 | >65 | MALE | G2 | Stage III | T3 | M0 | N2 |
| TCGA-RD-A8N0 | <=65 | FEMALE | G3 | Stage III | T3 | M0 | N2 |
| TCGA-RD-A8MV | <=65 | MALE | G3 | Stage III | T3 | M0 | N2 |
| TCGA-RD-A8N1 | >65 | MALE | G3 | Stage III | T3 | M0 | N2 |
| TCGA-R5-A805 | >65 | MALE | G3 | Stage III | T3 | M0 | N2 |
| TCGA-RD-A8MW | >65 | MALE | G3 | Stage III | T3 | M0 | N2 |
| TCGA-VQ-A94T | >65 | MALE | G3 | Stage III | T3 | M0 | N2 |
| TCGA-VQ-A91X | >65 | MALE | G3 | Stage III | T3 | M0 | N2 |
| TCGA-BR-4368 | >65 | FEMALE | G3 | Stage IV | T4 | M0 | N2 |
| TCGA-CG-4438 | <=65 | MALE | G3 | Stage IV | T4 | M0 | N2 |
| TCGA-BR-A4IZ | <=65 | FEMALE | G3 | Stage III | T4 | M0 | N2 |
| TCGA-BR-8058 | <=65 | FEMALE | G3 | Stage III | T4 | M0 | N2 |
| TCGA-BR-A4J4 | <=65 | MALE | G3 | Stage III | T4 | M0 | N2 |
| TCGA-BR-A4IV | <=65 | MALE | G3 | Stage III | T4 | M0 | N2 |
| TCGA-HU-8608 | >65 | MALE | G3 | Stage III | T4 | M0 | N2 |
| TCGA-BR-A4PF | >65 | MALE | G3 | Stage III | T4 | M0 | N2 |
| TCGA-HU-A4GC | >65 | MALE | G3 | Stage III | T4 | M0 | N2 |
| TCGA-BR-8683 | >65 | MALE | G3 | Stage III | T4 | M0 | N2 |
| TCGA-VQ-AA6J | >65 | MALE | G3 | Stage III | T4 | M0 | N2 |
| TCGA-VQ-A94R | <=65 | MALE | G2 | unknown | T4 | M0 | N2 |
| TCGA-VQ-A91D | >65 | MALE | G2 | Stage III | T4 | M0 | N2 |
| TCGA-HU-A4GQ | >65 | MALE | G2 | Stage III | T4 | M0 | N2 |
| TCGA-BR-8364 | <=65 | FEMALE | G3 | Stage III | T4 | M0 | N2 |
| TCGA-BR-8296 | <=65 | FEMALE | G3 | Stage III | T4 | M0 | N2 |
| TCGA-BR-8687 | >65 | FEMALE | G3 | Stage III | T4 | M0 | N2 |
| TCGA-HU-A4GX | >65 | FEMALE | G3 | Stage III | T4 | M0 | N2 |
| TCGA-BR-8361 | >65 | FEMALE | G3 | Stage III | T4 | M0 | N2 |
| TCGA-BR-8590 | <=65 | MALE | G3 | Stage III | T4 | M0 | N2 |
| TCGA-BR-8384 | >65 | MALE | G3 | Stage III | T4 | M0 | N2 |
| TCGA-CG-5722 | >65 | FEMALE | G3 | Stage IV | T3 | M1 | N2 |
| TCGA-R5-A804 | <=65 | MALE | G3 | Stage IV | T3 | M1 | N2 |
| TCGA-CG-4306 | >65 | MALE | G3 | Stage IV | T3 | M1 | N2 |
| TCGA-VQ-A8PJ | <=65 | MALE | unknown | Stage IV | T4 | M1 | N2 |
| TCGA-CG-5716 | >65 | MALE | G2 | Stage IV | T4 | M1 | N2 |
| TCGA-CG-4474 | >65 | FEMALE | G3 | Stage IV | T4 | M1 | N2 |
| TCGA-BR-8680 | <=65 | MALE | G2 | Stage IV | T4 | M1 | N2 |
| TCGA-FP-8631 | >65 | MALE | G2 | Stage III | T3 | unknown | N2 |
| TCGA-IP-7968 | >65 | MALE | G2 | Stage III | T3 | unknown | N2 |
| TCGA-3M-AB47 | <=65 | MALE | G3 | Stage III | T3 | unknown | N2 |
| TCGA-IN-A6RP | <=65 | MALE | G3 | unknown | T1 | M0 | N3 |
| TCGA-D7-5577 | <=65 | FEMALE | G2 | Stage III | T2 | M0 | N3 |
| TCGA-D7-6818 | <=65 | MALE | G3 | Stage III | T2 | M0 | N3 |
| TCGA-D7-6518 | >65 | MALE | G3 | Stage III | T2 | M0 | N3 |
| TCGA-CG-5732 | >65 | MALE | G2 | Stage IV | T2 | M0 | N3 |
| TCGA-D7-6817 | <=65 | MALE | G3 | Stage III | T2 | M0 | N3 |
| TCGA-HF-7133 | unknown | FEMALE | G2 | Stage IV | T2 | M0 | N3 |
| TCGA-VQ-A927 | >65 | MALE | G1 | Stage III | T3 | M0 | N3 |
| TCGA-VQ-A8PH | <=65 | MALE | G2 | Stage III | T3 | M0 | N3 |
| TCGA-D7-A74B | <=65 | FEMALE | G3 | Stage III | T3 | M0 | N3 |
| TCGA-VQ-AA6I | >65 | MALE | G3 | Stage III | T3 | M0 | N3 |
| TCGA-IN-A7NU | >65 | MALE | G3 | Stage III | T3 | M0 | N3 |
| TCGA-VQ-A928 | <=65 | MALE | G2 | Stage IV | T3 | M0 | N3 |
| TCGA-VQ-A91Q | <=65 | MALE | G3 | Stage IV | T3 | M0 | N3 |
| TCGA-RD-A7BT | >65 | MALE | G3 | Stage IV | T3 | M0 | N3 |
| TCGA-CG-4469 | >65 | MALE | G3 | Stage IV | T3 | M0 | N3 |
| TCGA-B7-A5TI | <=65 | MALE | G3 | Stage III | T4 | M0 | N3 |
| TCGA-VQ-A91N | <=65 | FEMALE | G2 | Stage IV | T4 | M0 | N3 |
| TCGA-VQ-A8PM | <=65 | MALE | G3 | Stage IV | T4 | M0 | N3 |
| TCGA-VQ-AA68 | <=65 | FEMALE | G2 | Stage III | T4 | M0 | N3 |
| TCGA-VQ-A8PD | >65 | MALE | G2 | Stage III | T4 | M0 | N3 |
| TCGA-VQ-A94O | >65 | MALE | G2 | Stage III | T4 | M0 | N3 |
| TCGA-IN-A7NR | <=65 | FEMALE | G3 | Stage IV | T3 | M1 | N3 |
| TCGA-CG-4440 | >65 | FEMALE | G3 | Stage IV | T3 | M1 | N3 |
| TCGA-CG-4462 | >65 | FEMALE | G3 | Stage IV | T3 | M1 | N3 |
| TCGA-CG-5724 | <=65 | MALE | G3 | Stage IV | T3 | M1 | N3 |
| TCGA-IN-7808 | <=65 | MALE | G3 | unknown | T3 | M1 | N3 |
| TCGA-EQ-5647 | >65 | FEMALE | G2 | Stage IV | T4 | M1 | N3 |
| TCGA-CG-4465 | >65 | FEMALE | G3 | Stage IV | T4 | M1 | N3 |
| TCGA-VQ-A8DZ | >65 | MALE | G3 | Stage IV | T4 | M1 | N3 |
| TCGA-D7-A748 | <=65 | FEMALE | G3 | Stage IV | T4 | M1 | N3 |
| TCGA-ZQ-A9CR | >65 | FEMALE | G3 | Stage III | T4 | unknown | N3 |
| TCGA-D7-A4YT | <=65 | MALE | G2 | Stage III | T2 | M0 | N3 |
| TCGA-D7-8574 | >65 | MALE | G3 | Stage III | T2 | M0 | N3 |
| TCGA-VQ-A91A | >65 | MALE | G1 | Stage III | T3 | M0 | N3 |
| TCGA-BR-8690 | <=65 | FEMALE | G2 | Stage III | T3 | M0 | N3 |
| TCGA-BR-A4QL | >65 | FEMALE | G2 | Stage III | T3 | M0 | N3 |
| TCGA-VQ-A8PK | <=65 | MALE | G2 | Stage III | T3 | M0 | N3 |
| TCGA-D7-A6ET | >65 | MALE | G2 | Stage III | T3 | M0 | N3 |
| TCGA-D7-8576 | <=65 | FEMALE | G3 | Stage III | T3 | M0 | N3 |
| TCGA-BR-6709 | <=65 | FEMALE | G3 | Stage III | T3 | M0 | N3 |
| TCGA-BR-6705 | >65 | FEMALE | G3 | Stage III | T3 | M0 | N3 |
| TCGA-BR-A4J8 | >65 | FEMALE | G3 | Stage III | T3 | M0 | N3 |
| TCGA-D7-A6EY | >65 | FEMALE | G3 | Stage III | T3 | M0 | N3 |
| TCGA-BR-8677 | >65 | FEMALE | G3 | Stage III | T3 | M0 | N3 |
| TCGA-BR-8369 | >65 | FEMALE | G3 | Stage III | T3 | M0 | N3 |
| TCGA-VQ-A8DT | <=65 | MALE | G3 | Stage III | T3 | M0 | N3 |
| TCGA-D7-8570 | <=65 | MALE | G3 | Stage III | T3 | M0 | N3 |
| TCGA-BR-8367 | <=65 | MALE | G3 | Stage III | T3 | M0 | N3 |
| TCGA-BR-8676 | <=65 | MALE | G3 | Stage III | T3 | M0 | N3 |
| TCGA-BR-7723 | <=65 | MALE | G3 | Stage III | T3 | M0 | N3 |
| TCGA-D7-A4YY | <=65 | MALE | G3 | Stage III | T3 | M0 | N3 |
| TCGA-BR-8371 | <=65 | MALE | G3 | Stage III | T3 | M0 | N3 |
| TCGA-BR-8370 | <=65 | MALE | G3 | Stage III | T3 | M0 | N3 |
| TCGA-BR-A44U | >65 | MALE | G3 | Stage III | T3 | M0 | N3 |
| TCGA-D7-A4YU | >65 | MALE | G3 | Stage III | T3 | M0 | N3 |
| TCGA-VQ-A8PE | >65 | MALE | G3 | Stage III | T3 | M0 | N3 |
| TCGA-VQ-AA6A | <=65 | MALE | G2 | Stage III | T4 | M0 | N3 |
| TCGA-HU-A4GJ | <=65 | FEMALE | G3 | Stage III | T4 | M0 | N3 |
| TCGA-BR-8485 | >65 | FEMALE | G3 | Stage III | T4 | M0 | N3 |
| TCGA-BR-8080 | >65 | FEMALE | G3 | Stage III | T4 | M0 | N3 |
| TCGA-BR-8285 | <=65 | FEMALE | G3 | Stage III | T4 | M0 | N3 |
| TCGA-BR-8284 | >65 | FEMALE | G3 | Stage III | T4 | M0 | N3 |
| TCGA-HF-A5NB | >65 | FEMALE | G3 | Stage III | T4 | M0 | N3 |
| TCGA-BR-8380 | <=65 | MALE | G3 | Stage III | T4 | M0 | N3 |
| TCGA-VQ-AA6K | <=65 | MALE | G3 | Stage III | T4 | M0 | N3 |
| TCGA-BR-8591 | >65 | MALE | G3 | Stage III | T4 | M0 | N3 |
| TCGA-BR-8382 | >65 | FEMALE | G3 | Stage III | T4 | M0 | N3 |
| TCGA-BR-A4CR | >65 | FEMALE | G3 | Stage III | T4 | M0 | N3 |
| TCGA-BR-8372 | <=65 | MALE | G3 | Stage III | T4 | M0 | N3 |
| TCGA-BR-8362 | <=65 | MALE | G3 | Stage III | T4 | M0 | N3 |
| TCGA-CG-4476 | >65 | MALE | G3 | Stage III | T4 | M0 | N3 |
| TCGA-BR-A4CS | >65 | MALE | G3 | Stage III | T4 | M0 | N3 |
| TCGA-BR-8297 | <=65 | MALE | unknown | Stage III | T4 | M0 | N3 |
| TCGA-BR-7196 | <=65 | MALE | G3 | Stage IV | T3 | M1 | N3 |
| TCGA-BR-7957 | <=65 | FEMALE | unknown | Stage IV | T3 | M1 | N3 |
| TCGA-BR-8289 | <=65 | MALE | G3 | Stage IV | T4 | M1 | N3 |
| TCGA-BR-A453 | <=65 | MALE | G3 | Stage IV | T4 | M1 | N3 |
| TCGA-FP-7916 | >65 | MALE | G3 | Stage III | T4 | unknown | N3 |
| TCGA-FP-7998 | >65 | MALE | G3 | Stage III | T4 | unknown | N3 |
| TCGA-FP-A8CX | <=65 | MALE | G3 | Stage III | T4 | unknown | N3 |
| TCGA-HU-A4H2 | <=65 | FEMALE | G2 | Stage III | T3 | M0 | N3 |
| TCGA-F1-A448 | >65 | MALE | G3 | Stage III | T3 | M0 | N3 |
| TCGA-VQ-A8PF | >65 | MALE | G3 | Stage III | T3 | M0 | N3 |
| TCGA-HU-8243 | >65 | MALE | G2 | Stage III | T4 | M0 | N3 |
| TCGA-HU-A4H3 | <=65 | FEMALE | G3 | Stage III | T4 | M0 | N3 |
| TCGA-VQ-A91Y | >65 | MALE | G3 | Stage III | T4 | M0 | N3 |
| TCGA-HU-A4H0 | >65 | MALE | G3 | Stage III | T4 | M0 | N3 |
| TCGA-BR-4292 | >65 | FEMALE | G1 | unknown | T1 | M0 | unknown |
| TCGA-BR-4371 | >65 | FEMALE | G1 | unknown | T2 | M0 | unknown |
| TCGA-BR-4201 | >65 | FEMALE | G1 | unknown | T2 | M0 | unknown |
| TCGA-BR-4294 | <=65 | MALE | G2 | unknown | T2 | M0 | unknown |
| TCGA-R5-A7ZR | >65 | FEMALE | G2 | Stage III | T3 | M0 | unknown |
| TCGA-B7-A5TJ | >65 | MALE | G1 | Stage II | T4 | M0 | unknown |
| TCGA-B7-A5TN | <=65 | MALE | G2 | Stage II | T4 | M0 | unknown |
| TCGA-BR-A4J7 | <=65 | MALE | G3 | Stage II | T4 | M0 | unknown |
| TCGA-BR-4370 | >65 | FEMALE | G2 | unknown | unknown | M0 | unknown |
| TCGA-BR-4257 | >65 | FEMALE | G2 | unknown | unknown | M0 | unknown |
| TCGA-BR-4366 | >65 | MALE | G2 | unknown | unknown | M0 | unknown |
| TCGA-BR-4187 | <=65 | MALE | G3 | unknown | unknown | M0 | unknown |
| TCGA-BR-4369 | >65 | MALE | G3 | unknown | unknown | M0 | unknown |
| TCGA-MX-A666 | <=65 | MALE | G2 | Stage II | T2 | unknown | unknown |
| TCGA-BR-4357 | <=65 | MALE | G2 | unknown | T3 | unknown | unknown |
| TCGA-BR-4363 | <=65 | FEMALE | G3 | unknown | T3 | unknown | unknown |
| TCGA-VQ-A94P | <=65 | MALE | unknown | unknown | T4 | unknown | unknown |
| TCGA-HU-8238 | <=65 | MALE | G2 | unknown | T3 | M0 | unknown |
| TCGA-B7-A5TK | <=65 | MALE | G3 | Stage III | T4 | M0 | unknown |

| GSE62254 | | | | | | | |
| --- | --- | --- | --- | --- | --- | --- | --- |
| id | Gender | Age | T stage | N stage | M stage | Stage | Lauren |
| GSM1523906 | M | <65 | 2 | 0 | 0 | I | diffuse |
| GSM1523858 | F | <65 | 2 | 0 | 0 | I | diffuse |
| GSM1523887 | M | <65 | 2 | 0 | 0 | I | diffuse |
| GSM1523885 | F | <65 | 2 | 0 | 0 | I | diffuse |
| GSM1523880 | F | <65 | 2 | 0 | 0 | I | diffuse |
| GSM1524059 | M | <65 | 3 | 0 | 0 | II | diffuse |
| GSM1523935 | M | <65 | 3 | 0 | 0 | II | diffuse |
| GSM1523984 | M | <65 | 2 | 1 | 0 | II | diffuse |
| GSM1523729 | F | <65 | 2 | 1 | 0 | II | diffuse |
| GSM1523921 | M | <65 | 2 | 1 | 0 | II | diffuse |
| GSM1523927 | M | <65 | 2 | 1 | 0 | II | diffuse |
| GSM1523776 | M | <65 | 2 | 1 | 0 | II | diffuse |
| GSM1524051 | F | <65 | 2 | 1 | 0 | II | diffuse |
| GSM1523856 | F | <65 | 2 | 1 | 0 | II | diffuse |
| GSM1523981 | M | <65 | 2 | 1 | 0 | II | diffuse |
| GSM1523924 | M | <65 | 2 | 1 | 0 | II | diffuse |
| GSM1523952 | F | <65 | 2 | 1 | 0 | II | diffuse |
| GSM1524046 | F | <65 | 2 | 1 | 0 | II | diffuse |
| GSM1523925 | F | <65 | 2 | 1 | 0 | II | diffuse |
| GSM1523923 | M | <65 | 2 | 1 | 0 | II | diffuse |
| GSM1523954 | M | <65 | 2 | 1 | 0 | II | diffuse |
| GSM1523977 | M | <65 | 2 | 1 | 0 | II | diffuse |
| GSM1523905 | M | ≥65 | 2 | 1 | 0 | II | diffuse |
| GSM1523869 | F | ≥65 | 2 | 1 | 0 | II | diffuse |
| GSM1523744 | M | ≥65 | 2 | 1 | 0 | II | diffuse |
| GSM1523854 | M | ≥65 | 2 | 1 | 0 | II | diffuse |
| GSM1523936 | M | ≥65 | 2 | 1 | 0 | II | diffuse |
| GSM1523842 | M | ≥65 | 2 | 1 | 0 | II | diffuse |
| GSM1524047 | M | ≥65 | 2 | 1 | 0 | II | diffuse |
| GSM1524052 | M | ≥65 | 2 | 1 | 0 | II | diffuse |
| GSM1523933 | M | ≥65 | 2 | 1 | 0 | II | diffuse |
| GSM1523909 | M | ≥65 | 2 | 1 | 0 | II | diffuse |
| GSM1523996 | F | ≥65 | 2 | 1 | 0 | II | diffuse |
| GSM1523775 | F | ≥65 | 2 | 1 | 0 | II | diffuse |
| GSM1523840 | M | ≥65 | 2 | 1 | 0 | II | diffuse |
| GSM1523793 | F | ≥65 | 2 | 1 | 0 | II | diffuse |
| GSM1523958 | M | <65 | 2 | 1 | 0 | II | diffuse |
| GSM1523931 | F | <65 | 2 | 1 | 0 | II | diffuse |
| GSM1523798 | M | ≥65 | 2 | 1 | 0 | II | diffuse |
| GSM1523804 | F | <65 | 3 | 1 | 0 | III | diffuse |
| GSM1524062 | F | <65 | 3 | 1 | 0 | III | diffuse |
| GSM1523971 | F | <65 | 3 | 1 | 0 | III | diffuse |
| GSM1524030 | F | <65 | 3 | 1 | 0 | III | diffuse |
| GSM1523884 | M | <65 | 3 | 1 | 0 | III | diffuse |
| GSM1524026 | F | <65 | 3 | 1 | 0 | III | diffuse |
| GSM1523910 | M | <65 | 3 | 1 | 0 | III | diffuse |
| GSM1524021 | F | <65 | 3 | 1 | 0 | III | diffuse |
| GSM1523808 | M | <65 | 3 | 1 | 0 | III | diffuse |
| GSM1524012 | M | <65 | 3 | 1 | 0 | III | diffuse |
| GSM1523748 | F | ≥65 | 3 | 1 | 0 | III | diffuse |
| GSM1523969 | M | ≥65 | 3 | 1 | 0 | III | diffuse |
| GSM1523813 | M | ≥65 | 3 | 1 | 0 | III | diffuse |
| GSM1523812 | M | <65 | 3 | 1 | 0 | III | diffuse |
| GSM1523975 | M | ≥65 | 3 | 1 | 0 | III | diffuse |
| GSM1523848 | M | ≥65 | 3 | 1 | 0 | III | diffuse |
| GSM1523827 | F | <65 | 3 | 1 | 0 | III | diffuse |
| GSM1523834 | F | ≥65 | 3 | 1 | 0 | III | diffuse |
| GSM1523784 | F | <65 | 2 | 2 | 0 | III | diffuse |
| GSM1523990 | M | <65 | 2 | 2 | 0 | III | diffuse |
| GSM1524006 | F | <65 | 2 | 2 | 0 | III | diffuse |
| GSM1523967 | M | <65 | 2 | 2 | 0 | III | diffuse |
| GSM1523783 | F | <65 | 2 | 2 | 0 | III | diffuse |
| GSM1524009 | F | <65 | 2 | 2 | 0 | III | diffuse |
| GSM1523983 | M | <65 | 2 | 2 | 0 | III | diffuse |
| GSM1524042 | F | <65 | 2 | 2 | 0 | III | diffuse |
| GSM1523973 | M | <65 | 2 | 2 | 0 | III | diffuse |
| GSM1523988 | M | ≥65 | 2 | 2 | 0 | III | diffuse |
| GSM1524025 | M | ≥65 | 2 | 2 | 0 | III | diffuse |
| GSM1523944 | M | ≥65 | 2 | 2 | 0 | III | diffuse |
| GSM1524010 | F | ≥65 | 2 | 2 | 0 | III | diffuse |
| GSM1523882 | M | <65 | 2 | 2 | 0 | III | diffuse |
| GSM1523846 | M | <65 | 2 | 2 | 0 | III | diffuse |
| GSM1523953 | M | ≥65 | 2 | 2 | 0 | III | diffuse |
| GSM1523987 | F | <65 | 3 | 2 | 0 | III | diffuse |
| GSM1523890 | F | <65 | 3 | 2 | 0 | III | diffuse |
| GSM1523962 | M | <65 | 3 | 2 | 0 | III | diffuse |
| GSM1523765 | F | <65 | 3 | 2 | 0 | III | diffuse |
| GSM1523778 | F | <65 | 3 | 2 | 0 | III | diffuse |
| GSM1523839 | M | <65 | 3 | 2 | 0 | III | diffuse |
| GSM1524027 | M | <65 | 3 | 2 | 0 | III | diffuse |
| GSM1523918 | M | <65 | 3 | 2 | 0 | III | diffuse |
| GSM1523745 | M | ≥65 | 3 | 2 | 0 | III | diffuse |
| GSM1524000 | F | ≥65 | 3 | 2 | 0 | III | diffuse |
| GSM1523986 | M | ≥65 | 3 | 2 | 0 | III | diffuse |
| GSM1524039 | M | ≥65 | 3 | 2 | 0 | III | diffuse |
| GSM1523980 | F | ≥65 | 3 | 2 | 0 | III | diffuse |
| GSM1523779 | F | ≥65 | 3 | 2 | 0 | III | diffuse |
| GSM1524024 | M | <65 | 3 | 2 | 0 | III | diffuse |
| GSM1523850 | M | <65 | 4 | 2 | 0 | IV | diffuse |
| GSM1523966 | M | <65 | 2 | 3 | 0 | IV | diffuse |
| GSM1523978 | F | ≥65 | 2 | 3 | 0 | IV | diffuse |
| GSM1523794 | M | ≥65 | 2 | 3 | 0 | IV | diffuse |
| GSM1523941 | M | ≥65 | 2 | 3 | 0 | IV | diffuse |
| GSM1523939 | F | <65 | 3 | 3 | 0 | IV | diffuse |
| GSM1524008 | M | <65 | 3 | 3 | 0 | IV | diffuse |
| GSM1523862 | M | <65 | 3 | 3 | 0 | IV | diffuse |
| GSM1523886 | M | <65 | 3 | 3 | 0 | IV | diffuse |
| GSM1523965 | M | <65 | 3 | 3 | 0 | IV | diffuse |
| GSM1523948 | M | <65 | 3 | 3 | 0 | IV | diffuse |
| GSM1523938 | F | <65 | 3 | 3 | 0 | IV | diffuse |
| GSM1524040 | F | <65 | 3 | 3 | 0 | IV | diffuse |
| GSM1524022 | F | ≥65 | 3 | 3 | 0 | IV | diffuse |
| GSM1523833 | M | ≥65 | 3 | 3 | 0 | IV | diffuse |
| GSM1523818 | F | ≥65 | 3 | 3 | 0 | IV | diffuse |
| GSM1523992 | F | ≥65 | 3 | 3 | 0 | IV | diffuse |
| GSM1524019 | M | ≥65 | 3 | 3 | 0 | IV | diffuse |
| GSM1523769 | F | ≥65 | 3 | 3 | 0 | IV | diffuse |
| GSM1523817 | M | ≥65 | 3 | 3 | 0 | IV | diffuse |
| GSM1523773 | F | ≥65 | 3 | 3 | 0 | IV | diffuse |
| GSM1523845 | F | ≥65 | 3 | 3 | 0 | IV | diffuse |
| GSM1523908 | F | <65 | 4 | 3 | 0 | IV | diffuse |
| GSM1524029 | F | <65 | 4 | 3 | 0 | IV | diffuse |
| GSM1523945 | M | <65 | 4 | 3 | 0 | IV | diffuse |
| GSM1523997 | F | ≥65 | 4 | 3 | 0 | IV | diffuse |
| GSM1523968 | F | <65 | 3 | 0 | 1 | IV | diffuse |
| GSM1523831 | M | ≥65 | 2 | 1 | 1 | IV | diffuse |
| GSM1524007 | F | <65 | 3 | 1 | 1 | IV | diffuse |
| GSM1523772 | F | <65 | 3 | 1 | 1 | IV | diffuse |
| GSM1523873 | M | <65 | 2 | 2 | 1 | IV | diffuse |
| GSM1524043 | M | <65 | 2 | 2 | 1 | IV | diffuse |
| GSM1524072 | F | <65 | 3 | 2 | 1 | IV | diffuse |
| GSM1523949 | F | <65 | 3 | 2 | 1 | IV | diffuse |
| GSM1523982 | M | <65 | 3 | 2 | 1 | IV | diffuse |
| GSM1523943 | F | ≥65 | 3 | 2 | 1 | IV | diffuse |
| GSM1523771 | F | <65 | 4 | 2 | 1 | IV | diffuse |
| GSM1523843 | F | ≥65 | 4 | 2 | 1 | IV | diffuse |
| GSM1523951 | M | ≥65 | 4 | 2 | 1 | IV | diffuse |
| GSM1523788 | F | <65 | 2 | 3 | 1 | IV | diffuse |
| GSM1523786 | F | <65 | 2 | 3 | 1 | IV | diffuse |
| GSM1523789 | M | ≥65 | 2 | 3 | 1 | IV | diffuse |
| GSM1524038 | M | ≥65 | 2 | 3 | 1 | IV | diffuse |
| GSM1524016 | M | <65 | 3 | 3 | 1 | IV | diffuse |
| GSM1523872 | F | <65 | 4 | 3 | 1 | IV | diffuse |
| GSM1524004 | M | <65 | 4 | 3 | 1 | IV | diffuse |
| GSM1523801 | M | ≥65 | 2 | 1 | 0 | II | indeterminate |
| GSM1523803 | F | ≥65 | 3 | 3 | 0 | IV | indeterminate |
| GSM1523864 | M | <65 | 2 | 0 | 0 | I | intestinal |
| GSM1523888 | M | <65 | 2 | 0 | 0 | I | intestinal |
| GSM1523891 | F | <65 | 2 | 0 | 0 | I | intestinal |
| GSM1523792 | M | <65 | 2 | 0 | 0 | I | intestinal |
| GSM1523894 | M | <65 | 2 | 0 | 0 | I | intestinal |
| GSM1523892 | M | <65 | 2 | 0 | 0 | I | intestinal |
| GSM1523791 | M | <65 | 2 | 0 | 0 | I | intestinal |
| GSM1523898 | M | <65 | 2 | 0 | 0 | I | intestinal |
| GSM1523861 | M | <65 | 2 | 0 | 0 | I | intestinal |
| GSM1523901 | F | ≥65 | 2 | 0 | 0 | I | intestinal |
| GSM1523810 | M | ≥65 | 2 | 0 | 0 | I | intestinal |
| GSM1523822 | M | ≥65 | 2 | 0 | 0 | I | intestinal |
| GSM1523895 | M | ≥65 | 2 | 0 | 0 | I | intestinal |
| GSM1523853 | M | ≥65 | 2 | 0 | 0 | I | intestinal |
| GSM1523795 | M | ≥65 | 2 | 0 | 0 | I | intestinal |
| GSM1523837 | M | ≥65 | 2 | 0 | 0 | I | intestinal |
| GSM1523874 | F | ≥65 | 2 | 0 | 0 | I | intestinal |
| GSM1523889 | M | ≥65 | 2 | 0 | 0 | I | intestinal |
| GSM1523893 | M | ≥65 | 2 | 0 | 0 | I | intestinal |
| GSM1523876 | M | ≥65 | 2 | 0 | 0 | I | intestinal |
| GSM1523851 | F | ≥65 | 2 | 0 | 0 | I | intestinal |
| GSM1523897 | F | ≥65 | 2 | 0 | 0 | I | intestinal |
| GSM1523811 | F | ≥65 | 2 | 0 | 0 | I | intestinal |
| GSM1523829 | M | ≥65 | 2 | 0 | 0 | II | intestinal |
| GSM1523920 | M | <65 | 3 | 0 | 0 | II | intestinal |
| GSM1523947 | F | ≥65 | 3 | 0 | 0 | II | intestinal |
| GSM1523991 | F | <65 | 2 | 1 | 0 | II | intestinal |
| GSM1523932 | F | ≥65 | 2 | 1 | 0 | II | intestinal |
| GSM1523728 | F | ≥65 | 2 | 1 | 0 | II | intestinal |
| GSM1523807 | F | ≥65 | 2 | 1 | 0 | II | intestinal |
| GSM1523809 | M | ≥65 | 2 | 1 | 0 | II | intestinal |
| GSM1523937 | M | ≥65 | 2 | 1 | 0 | II | intestinal |
| GSM1523847 | F | <65 | 2 | 1 | 0 | II | intestinal |
| GSM1524045 | M | <65 | 2 | 1 | 0 | II | intestinal |
| GSM1523930 | M | <65 | 2 | 1 | 0 | II | intestinal |
| GSM1524055 | M | <65 | 2 | 1 | 0 | II | intestinal |
| GSM1523835 | M | <65 | 2 | 1 | 0 | II | intestinal |
| GSM1524058 | F | <65 | 2 | 1 | 0 | II | intestinal |
| GSM1524060 | M | <65 | 2 | 1 | 0 | II | intestinal |
| GSM1524070 | M | <65 | 2 | 1 | 0 | II | intestinal |
| GSM1524001 | M | <65 | 2 | 1 | 0 | II | intestinal |
| GSM1523899 | F | <65 | 2 | 1 | 0 | II | intestinal |
| GSM1524053 | M | <65 | 2 | 1 | 0 | II | intestinal |
| GSM1523964 | M | <65 | 2 | 1 | 0 | II | intestinal |
| GSM1523974 | M | <65 | 2 | 1 | 0 | II | intestinal |
| GSM1524049 | M | <65 | 2 | 1 | 0 | II | intestinal |
| GSM1523790 | M | <65 | 2 | 1 | 0 | II | intestinal |
| GSM1524011 | F | <65 | 2 | 1 | 0 | II | intestinal |
| GSM1524069 | M | <65 | 2 | 1 | 0 | II | intestinal |
| GSM1523961 | M | <65 | 2 | 1 | 0 | II | intestinal |
| GSM1523770 | M | <65 | 2 | 1 | 0 | II | intestinal |
| GSM1523824 | M | <65 | 2 | 1 | 0 | II | intestinal |
| GSM1523922 | M | ≥65 | 2 | 1 | 0 | II | intestinal |
| GSM1524050 | M | ≥65 | 2 | 1 | 0 | II | intestinal |
| GSM1523999 | M | ≥65 | 2 | 1 | 0 | II | intestinal |
| GSM1524048 | M | ≥65 | 2 | 1 | 0 | II | intestinal |
| GSM1523727 | M | ≥65 | 2 | 1 | 0 | II | intestinal |
| GSM1523859 | M | ≥65 | 2 | 1 | 0 | II | intestinal |
| GSM1523814 | M | ≥65 | 2 | 1 | 0 | II | intestinal |
| GSM1523963 | F | ≥65 | 2 | 1 | 0 | II | intestinal |
| GSM1523928 | M | ≥65 | 2 | 1 | 0 | II | intestinal |
| GSM1523960 | F | ≥65 | 2 | 1 | 0 | II | intestinal |
| GSM1523802 | F | ≥65 | 2 | 1 | 0 | II | intestinal |
| GSM1523871 | F | ≥65 | 2 | 1 | 0 | II | intestinal |
| GSM1523865 | M | ≥65 | 2 | 1 | 0 | II | intestinal |
| GSM1524068 | F | ≥65 | 2 | 1 | 0 | II | intestinal |
| GSM1524056 | M | ≥65 | 2 | 1 | 0 | II | intestinal |
| GSM1524054 | M | ≥65 | 2 | 1 | 0 | II | intestinal |
| GSM1523870 | M | ≥65 | 2 | 1 | 0 | II | intestinal |
| GSM1524061 | M | ≥65 | 2 | 1 | 0 | II | intestinal |
| GSM1523875 | M | ≥65 | 2 | 1 | 0 | II | intestinal |
| GSM1523896 | M | <65 | 2 | 1 | 0 | II | intestinal |
| GSM1523878 | M | <65 | 2 | 1 | 0 | II | intestinal |
| GSM1523957 | F | <65 | 2 | 1 | 0 | II | intestinal |
| GSM1523830 | M | <65 | 2 | 1 | 0 | II | intestinal |
| GSM1523785 | F | <65 | 2 | 1 | 0 | II | intestinal |
| GSM1524013 | M | <65 | 2 | 1 | 0 | II | intestinal |
| GSM1523816 | M | ≥65 | 2 | 1 | 0 | II | intestinal |
| GSM1523799 | M | ≥65 | 2 | 1 | 0 | II | intestinal |
| GSM1523903 | M | <65 | 4 | 0 | 0 | III | intestinal |
| GSM1523796 | M | <65 | 3 | 1 | 0 | III | intestinal |
| GSM1523852 | M | <65 | 3 | 1 | 0 | III | intestinal |
| GSM1523836 | M | <65 | 3 | 1 | 0 | III | intestinal |
| GSM1523820 | M | <65 | 3 | 1 | 0 | III | intestinal |
| GSM1524034 | M | ≥65 | 3 | 1 | 0 | III | intestinal |
| GSM1523849 | M | ≥65 | 3 | 1 | 0 | III | intestinal |
| GSM1524036 | M | ≥65 | 3 | 1 | 0 | III | intestinal |
| GSM1523826 | M | ≥65 | 3 | 1 | 0 | III | intestinal |
| GSM1524071 | M | <65 | 4 | 1 | 0 | III | intestinal |
| GSM1523946 | M | <65 | 2 | 2 | 0 | III | intestinal |
| GSM1524020 | M | <65 | 2 | 2 | 0 | III | intestinal |
| GSM1523768 | M | <65 | 2 | 2 | 0 | III | intestinal |
| GSM1523867 | M | <65 | 2 | 2 | 0 | III | intestinal |
| GSM1523774 | M | <65 | 2 | 2 | 0 | III | intestinal |
| GSM1523998 | M | <65 | 2 | 2 | 0 | III | intestinal |
| GSM1524003 | F | <65 | 2 | 2 | 0 | III | intestinal |
| GSM1523832 | M | <65 | 2 | 2 | 0 | III | intestinal |
| GSM1523994 | M | <65 | 2 | 2 | 0 | III | intestinal |
| GSM1523823 | F | <65 | 2 | 2 | 0 | III | intestinal |
| GSM1523877 | M | ≥65 | 2 | 2 | 0 | III | intestinal |
| GSM1523929 | F | ≥65 | 2 | 2 | 0 | III | intestinal |
| GSM1524017 | M | ≥65 | 2 | 2 | 0 | III | intestinal |
| GSM1523993 | M | ≥65 | 2 | 2 | 0 | III | intestinal |
| GSM1524028 | M | ≥65 | 2 | 2 | 0 | III | intestinal |
| GSM1523800 | M | ≥65 | 2 | 2 | 0 | III | intestinal |
| GSM1523881 | M | ≥65 | 2 | 2 | 0 | III | intestinal |
| GSM1523855 | M | ≥65 | 2 | 2 | 0 | III | intestinal |
| GSM1523985 | M | ≥65 | 2 | 2 | 0 | III | intestinal |
| GSM1523844 | M | <65 | 3 | 2 | 0 | III | intestinal |
| GSM1523797 | M | <65 | 3 | 2 | 0 | III | intestinal |
| GSM1523838 | M | ≥65 | 3 | 2 | 0 | III | intestinal |
| GSM1523806 | M | ≥65 | 3 | 2 | 0 | III | intestinal |
| GSM1523825 | M | ≥65 | 3 | 2 | 0 | III | intestinal |
| GSM1524037 | M | ≥65 | 3 | 2 | 0 | III | intestinal |
| GSM1523857 | F | ≥65 | 3 | 2 | 0 | III | intestinal |
| GSM1523979 | M | ≥65 | 3 | 2 | 0 | III | intestinal |
| GSM1524057 | M | ≥65 | 4 | 0 | 0 | IV | intestinal |
| GSM1524015 | M | <65 | 4 | 1 | 0 | IV | intestinal |
| GSM1523919 | M | <65 | 4 | 1 | 0 | IV | intestinal |
| GSM1523821 | M | <65 | 4 | 1 | 0 | IV | intestinal |
| GSM1524032 | F | ≥65 | 4 | 1 | 0 | IV | intestinal |
| GSM1523819 | F | ≥65 | 4 | 1 | 0 | IV | intestinal |
| GSM1523780 | F | ≥65 | 4 | 1 | 0 | IV | intestinal |
| GSM1524023 | F | ≥65 | 4 | 2 | 0 | IV | intestinal |
| GSM1523781 | F | <65 | 2 | 3 | 0 | IV | intestinal |
| GSM1523959 | M | <65 | 2 | 3 | 0 | IV | intestinal |
| GSM1524031 | M | <65 | 2 | 3 | 0 | IV | intestinal |
| GSM1523904 | M | <65 | 2 | 3 | 0 | IV | intestinal |
| GSM1523787 | F | <65 | 2 | 3 | 0 | IV | intestinal |
| GSM1523950 | M | <65 | 2 | 3 | 0 | IV | intestinal |
| GSM1523777 | M | <65 | 2 | 3 | 0 | IV | intestinal |
| GSM1523782 | M | <65 | 2 | 3 | 0 | IV | intestinal |
| GSM1523989 | M | <65 | 3 | 3 | 0 | IV | intestinal |
| GSM1523879 | M | <65 | 3 | 3 | 0 | IV | intestinal |
| GSM1523940 | M | ≥65 | 3 | 3 | 0 | IV | intestinal |
| GSM1523934 | M | ≥65 | 3 | 3 | 0 | IV | intestinal |
| GSM1523911 | F | ≥65 | 3 | 3 | 0 | IV | intestinal |
| GSM1523828 | M | ≥65 | 3 | 3 | 0 | IV | intestinal |
| GSM1524044 | M | ≥65 | 2 | 1 | 1 | IV | intestinal |
| GSM1524033 | F | ≥65 | 2 | 2 | 1 | IV | intestinal |
| GSM1523866 | F | ≥65 | 3 | 2 | 1 | IV | intestinal |
| GSM1524041 | M | <65 | 2 | 3 | 1 | IV | intestinal |
| GSM1524005 | M | ≥65 | 2 | 3 | 1 | IV | intestinal |
| GSM1524014 | F | ≥65 | 4 | 3 | 1 | IV | intestinal |
| GSM1523863 | F | <65 | 2 | 0 | 0 | I | mixed |
| GSM1523860 | M | ≥65 | 2 | 0 | 0 | I | mixed |
| GSM1523746 | M | <65 | 2 | 1 | 0 | II | mixed |
| GSM1523956 | M | ≥65 | 2 | 1 | 0 | II | mixed |
| GSM1523970 | F | ≥65 | 2 | 1 | 0 | II | mixed |
| GSM1523841 | M | ≥65 | 2 | 1 | 0 | II | mixed |
| GSM1524018 | M | <65 | 3 | 1 | 0 | III | mixed |
| GSM1523976 | M | ≥65 | 2 | 2 | 0 | III | mixed |
| GSM1523955 | M | <65 | 2 | 2 | 0 | III | mixed |
| GSM1523747 | M | <65 | 2 | 2 | 0 | III | mixed |
| GSM1523815 | M | <65 | 2 | 2 | 0 | III | mixed |
| GSM1523995 | M | ≥65 | 2 | 2 | 0 | III | mixed |
| GSM1523868 | M | <65 | 3 | 2 | 0 | III | mixed |
| GSM1524002 | M | <65 | 3 | 2 | 0 | III | mixed |
| GSM1523805 | F | ≥65 | 3 | 2 | 0 | III | mixed |
| GSM1524035 | F | <65 | 3 | 3 | 0 | IV | mixed |
| GSM1523972 | M | ≥65 | 2 | 1 | 1 | IV | mixed |

| GSE84437 | | | | |
| --- | --- | --- | --- | --- |
| Id | Age | Gender | T stage | N stage |
| GSM2235556 | <65 | male | T3 | N1 |
| GSM2235557 | <65 | female | T4 | N3 |
| GSM2235558 | ≥65 | male | T4 | N1 |
| GSM2235559 | ≥65 | male | T3 | N2 |
| GSM2235560 | <65 | male | T4 | N0 |
| GSM2235561 | <65 | female | T4 | N2 |
| GSM2235562 | ≥65 | female | T4 | N2 |
| GSM2235563 | <65 | female | T3 | N2 |
| GSM2235564 | <65 | male | T3 | N1 |
| GSM2235565 | <65 | female | T4 | N0 |
| GSM2235566 | <65 | male | T4 | N2 |
| GSM2235567 | <65 | male | T4 | N2 |
| GSM2235568 | <65 | female | T3 | N1 |
| GSM2235569 | ≥65 | male | T4 | N1 |
| GSM2235570 | ≥65 | male | T4 | N0 |
| GSM2235571 | ≥65 | female | T3 | N1 |
| GSM2235572 | ≥65 | female | T4 | N1 |
| GSM2235573 | <65 | male | T4 | N2 |
| GSM2235574 | ≥65 | male | T4 | N2 |
| GSM2235575 | <65 | male | T3 | N1 |
| GSM2235576 | ≥65 | female | T3 | N2 |
| GSM2235577 | <65 | female | T4 | N0 |
| GSM2235578 | ≥65 | male | T4 | N2 |
| GSM2235579 | ≥65 | male | T3 | N1 |
| GSM2235580 | <65 | male | T4 | N2 |
| GSM2235582 | ≥65 | male | T4 | N0 |
| GSM2235584 | ≥65 | male | T4 | N2 |
| GSM2235585 | ≥65 | male | T3 | N1 |
| GSM2235586 | ≥65 | male | T4 | N1 |
| GSM2235587 | ≥65 | male | T4 | N1 |
| GSM2235588 | <65 | male | T4 | N1 |
| GSM2235589 | ≥65 | male | T4 | N2 |
| GSM2235593 | <65 | male | T4 | N0 |
| GSM2235595 | ≥65 | male | T4 | N1 |
| GSM2235596 | <65 | male | T3 | N1 |
| GSM2235597 | ≥65 | male | T2 | N1 |
| GSM2235598 | ≥65 | male | T3 | N1 |
| GSM2235599 | <65 | male | T3 | N2 |
| GSM2235600 | <65 | male | T4 | N2 |
| GSM2235601 | ≥65 | male | T4 | N2 |
| GSM2235602 | <65 | female | T3 | N1 |
| GSM2235603 | <65 | female | T4 | N1 |
| GSM2235604 | ≥65 | male | T3 | N1 |
| GSM2235605 | <65 | female | T4 | N1 |
| GSM2235606 | <65 | male | T4 | N2 |
| GSM2235607 | ≥65 | male | T3 | N2 |
| GSM2235608 | <65 | male | T3 | N1 |
| GSM2235609 | ≥65 | female | T4 | N2 |
| GSM2235610 | ≥65 | male | T4 | N2 |
| GSM2235611 | ≥65 | female | T4 | N2 |
| GSM2235612 | ≥65 | male | T4 | N2 |
| GSM2235613 | ≥65 | male | T2 | N1 |
| GSM2235614 | <65 | male | T4 | N2 |
| GSM2235615 | ≥65 | male | T3 | N2 |
| GSM2235616 | ≥65 | male | T4 | N1 |
| GSM2235617 | <65 | male | T4 | N2 |
| GSM2235618 | ≥65 | female | T4 | N2 |
| GSM2235619 | <65 | female | T4 | N1 |
| GSM2235620 | ≥65 | male | T3 | N2 |
| GSM2235621 | <65 | male | T4 | N2 |
| GSM2235622 | <65 | male | T3 | N2 |
| GSM2235623 | <65 | male | T3 | N1 |
| GSM2235624 | <65 | male | T3 | N1 |
| GSM2235625 | <65 | female | T4 | N0 |
| GSM2235626 | <65 | male | T4 | N2 |
| GSM2235627 | ≥65 | male | T3 | N1 |
| GSM2235628 | <65 | male | T3 | N1 |
| GSM2235629 | ≥65 | male | T4 | N1 |
| GSM2235630 | ≥65 | male | T4 | N1 |
| GSM2235631 | ≥65 | female | T4 | N0 |
| GSM2235632 | <65 | male | T3 | N1 |
| GSM2235633 | <65 | male | T4 | N2 |
| GSM2235634 | <65 | female | T4 | N1 |
| GSM2235635 | ≥65 | male | T2 | N2 |
| GSM2235636 | <65 | female | T4 | N2 |
| GSM2235637 | ≥65 | female | T4 | N0 |
| GSM2235695 | <65 | female | T4 | N1 |
| GSM2235696 | <65 | male | T4 | N1 |
| GSM2235697 | <65 | male | T3 | N0 |
| GSM2235698 | <65 | male | T3 | N3 |
| GSM2235699 | ≥65 | male | T4 | N3 |
| GSM2235700 | <65 | male | T4 | N2 |
| GSM2235701 | <65 | male | T3 | N0 |
| GSM2235702 | ≥65 | female | T4 | N1 |
| GSM2235703 | <65 | male | T4 | N2 |
| GSM2235704 | <65 | female | T4 | N1 |
| GSM2235705 | ≥65 | male | T4 | N1 |
| GSM2235706 | ≥65 | male | T4 | N2 |
| GSM2235707 | <65 | female | T2 | N1 |
| GSM2235708 | ≥65 | male | T4 | N1 |
| GSM2235709 | <65 | female | T2 | N0 |
| GSM2235710 | <65 | male | T4 | N0 |
| GSM2235711 | ≥65 | male | T4 | N1 |
| GSM2235712 | ≥65 | female | T2 | N0 |
| GSM2235713 | <65 | male | T4 | N0 |
| GSM2235714 | <65 | male | T2 | N1 |
| GSM2235715 | <65 | male | T4 | N1 |
| GSM2235716 | <65 | male | T3 | N1 |
| GSM2235717 | <65 | female | T4 | N3 |
| GSM2235718 | <65 | male | T4 | N1 |
| GSM2235719 | ≥65 | male | T4 | N2 |
| GSM2235720 | <65 | male | T1 | N0 |
| GSM2235721 | <65 | male | T2 | N1 |
| GSM2235722 | <65 | female | T4 | N2 |
| GSM2235723 | <65 | male | T3 | N2 |
| GSM2235724 | ≥65 | male | T3 | N3 |
| GSM2235725 | ≥65 | female | T4 | N0 |
| GSM2235726 | ≥65 | male | T4 | N2 |
| GSM2235727 | ≥65 | male | T4 | N3 |
| GSM2235728 | <65 | female | T4 | N1 |
| GSM2235729 | ≥65 | male | T4 | N2 |
| GSM2235730 | ≥65 | female | T1 | N1 |
| GSM2235731 | <65 | female | T2 | N1 |
| GSM2235732 | ≥65 | male | T2 | N1 |
| GSM2235733 | ≥65 | male | T3 | N1 |
| GSM2235734 | <65 | female | T4 | N1 |
| GSM2235735 | <65 | male | T3 | N2 |
| GSM2235736 | <65 | male | T4 | N2 |
| GSM2235737 | <65 | male | T4 | N2 |
| GSM2235738 | <65 | female | T4 | N2 |
| GSM2235739 | ≥65 | male | T3 | N2 |
| GSM2235740 | ≥65 | male | T3 | N1 |
| GSM2235741 | <65 | male | T4 | N2 |
| GSM2235742 | <65 | male | T4 | N2 |
| GSM2235743 | <65 | female | T4 | N1 |
| GSM2235744 | <65 | female | T4 | N1 |
| GSM2235745 | <65 | male | T4 | N2 |
| GSM2235746 | <65 | male | T3 | N1 |
| GSM2235747 | <65 | female | T4 | N2 |
| GSM2235748 | ≥65 | male | T4 | N2 |
| GSM2235749 | ≥65 | male | T4 | N3 |
| GSM2235750 | ≥65 | male | T4 | N1 |
| GSM2235751 | <65 | male | T3 | N1 |
| GSM2235752 | <65 | male | T4 | N1 |
| GSM2235753 | ≥65 | male | T4 | N1 |
| GSM2235754 | <65 | female | T4 | N1 |
| GSM2235755 | <65 | female | T4 | N1 |
| GSM2235756 | <65 | female | T4 | N2 |
| GSM2235757 | <65 | male | T4 | N2 |
| GSM2235758 | <65 | male | T4 | N2 |
| GSM2235759 | <65 | male | T4 | N2 |
| GSM2235760 | <65 | male | T4 | N2 |
| GSM2235761 | <65 | female | T4 | N1 |
| GSM2235762 | ≥65 | male | T4 | N2 |
| GSM2235763 | ≥65 | female | T4 | N1 |
| GSM2235764 | <65 | male | T4 | N2 |
| GSM2235765 | ≥65 | male | T4 | N1 |
| GSM2235766 | <65 | male | T4 | N2 |
| GSM2235767 | <65 | male | T4 | N2 |
| GSM2235768 | <65 | male | T4 | N1 |
| GSM2235769 | <65 | male | T3 | N2 |
| GSM2235770 | ≥65 | male | T3 | N1 |
| GSM2235771 | <65 | male | T4 | N1 |
| GSM2235772 | <65 | male | T4 | N2 |
| GSM2235773 | <65 | male | T4 | N2 |
| GSM2235774 | <65 | male | T4 | N1 |
| GSM2235775 | <65 | male | T4 | N1 |
| GSM2235776 | ≥65 | male | T4 | N0 |
| GSM2235777 | <65 | female | T4 | N1 |
| GSM2235778 | <65 | male | T4 | N3 |
| GSM2235779 | <65 | male | T4 | N0 |
| GSM2235780 | ≥65 | male | T4 | N3 |
| GSM2235781 | <65 | male | T1 | N0 |
| GSM2235782 | <65 | male | T4 | N2 |
| GSM2235783 | ≥65 | male | T3 | N1 |
| GSM2235784 | ≥65 | male | T2 | N0 |
| GSM2235785 | <65 | female | T3 | N0 |
| GSM2235786 | <65 | female | T4 | N1 |
| GSM2235787 | <65 | female | T4 | N2 |
| GSM2235788 | <65 | male | T2 | N0 |
| GSM2235789 | <65 | male | T4 | N2 |
| GSM2235790 | ≥65 | female | T4 | N0 |
| GSM2235791 | <65 | female | T4 | N1 |
| GSM2235792 | <65 | female | T4 | N1 |
| GSM2235793 | ≥65 | male | T3 | N2 |
| GSM2235794 | <65 | male | T1 | N1 |
| GSM2235795 | ≥65 | female | T4 | N0 |
| GSM2235796 | <65 | male | T4 | N3 |
| GSM2235797 | ≥65 | female | T3 | N0 |
| GSM2235798 | ≥65 | male | T4 | N2 |
| GSM2235799 | <65 | male | T4 | N3 |
| GSM2235800 | <65 | male | T3 | N1 |
| GSM2235801 | <65 | male | T2 | N1 |
| GSM2235802 | <65 | male | T1 | N0 |
| GSM2235803 | <65 | male | T3 | N0 |
| GSM2235804 | <65 | male | T4 | N0 |
| GSM2235805 | <65 | female | T4 | N1 |
| GSM2235806 | <65 | male | T4 | N0 |
| GSM2235807 | ≥65 | male | T4 | N3 |
| GSM2235808 | <65 | male | T4 | N1 |
| GSM2235809 | <65 | male | T4 | N1 |
| GSM2235810 | <65 | male | T4 | N1 |
| GSM2235811 | <65 | male | T4 | N2 |
| GSM2235812 | ≥65 | male | T4 | N3 |
| GSM2235813 | <65 | male | T3 | N0 |
| GSM2235814 | ≥65 | male | T4 | N3 |
| GSM2235815 | ≥65 | female | T4 | N2 |
| GSM2235816 | <65 | male | T4 | N3 |
| GSM2235817 | ≥65 | female | T4 | N1 |
| GSM2235818 | ≥65 | female | T4 | N3 |
| GSM2235819 | ≥65 | male | T4 | N1 |
| GSM2235820 | ≥65 | male | T4 | N1 |
| GSM2235821 | ≥65 | male | T4 | N1 |
| GSM2235822 | <65 | male | T3 | N1 |
| GSM2235823 | <65 | female | T4 | N1 |
| GSM2235824 | <65 | female | T4 | N2 |
| GSM2235825 | ≥65 | male | T4 | N0 |
| GSM2235826 | ≥65 | male | T3 | N2 |
| GSM2235827 | <65 | male | T4 | N2 |
| GSM2235828 | <65 | male | T4 | N1 |
| GSM2235829 | <65 | male | T4 | N0 |
| GSM2235830 | ≥65 | male | T4 | N2 |
| GSM2235831 | ≥65 | male | T4 | N3 |
| GSM2235837 | <65 | female | T2 | N0 |
| GSM2235838 | ≥65 | male | T4 | N3 |
| GSM2235839 | <65 | male | T3 | N1 |
| GSM2235840 | ≥65 | male | T2 | N0 |
| GSM2235841 | ≥65 | female | T4 | N1 |
| GSM2235842 | ≥65 | male | T4 | N3 |
| GSM2235843 | <65 | male | T2 | N1 |
| GSM2235844 | <65 | female | T4 | N2 |
| GSM2235845 | <65 | male | T4 | N2 |
| GSM2235846 | ≥65 | male | T4 | N2 |
| GSM2235847 | <65 | female | T4 | N1 |
| GSM2235848 | <65 | male | T4 | N2 |
| GSM2235849 | <65 | female | T4 | N2 |
| GSM2235850 | <65 | male | T2 | N1 |
| GSM2235851 | ≥65 | male | T1 | N0 |
| GSM2235852 | ≥65 | male | T4 | N3 |
| GSM2235853 | <65 | female | T2 | N1 |
| GSM2235854 | ≥65 | female | T4 | N3 |
| GSM2235855 | ≥65 | male | T2 | N1 |
| GSM2235862 | ≥65 | male | T4 | N0 |
| GSM2235863 | ≥65 | male | T4 | N0 |
| GSM2235864 | <65 | male | T4 | N1 |
| GSM2235865 | <65 | male | T4 | N1 |
| GSM2235866 | ≥65 | female | T4 | N2 |
| GSM2235867 | ≥65 | female | T4 | N2 |
| GSM2235878 | <65 | female | T4 | N3 |
| GSM2235879 | ≥65 | female | T3 | N1 |
| GSM2235880 | ≥65 | female | T4 | N2 |
| GSM2235881 | ≥65 | male | T4 | N0 |
| GSM2235882 | <65 | male | T4 | N1 |
| GSM2235883 | ≥65 | female | T4 | N2 |
| GSM2235884 | <65 | male | T4 | N0 |
| GSM2235885 | ≥65 | female | T4 | N1 |
| GSM2235886 | ≥65 | female | T2 | N1 |
| GSM2235887 | ≥65 | male | T3 | N2 |
| GSM2235888 | <65 | female | T4 | N1 |
| GSM2235889 | ≥65 | male | T4 | N1 |
| GSM2235899 | <65 | male | T4 | N1 |
| GSM2235900 | <65 | male | T4 | N1 |
| GSM2235901 | ≥65 | female | T3 | N1 |
| GSM2235902 | <65 | male | T4 | N1 |
| GSM2235903 | <65 | male | T4 | N1 |
| GSM2235904 | <65 | male | T4 | N1 |
| GSM2235905 | <65 | male | T4 | N1 |
| GSM2235906 | ≥65 | female | T4 | N1 |
| GSM2235907 | ≥65 | female | T4 | N0 |
| GSM2235908 | <65 | female | T3 | N0 |
| GSM2235909 | <65 | male | T4 | N2 |
| GSM2235910 | <65 | male | T4 | N1 |
| GSM2235921 | <65 | male | T2 | N2 |
| GSM2235922 | ≥65 | male | T4 | N1 |
| GSM2235923 | ≥65 | female | T4 | N1 |
| GSM2235924 | <65 | male | T4 | N2 |
| GSM2235925 | <65 | male | T4 | N1 |
| GSM2235926 | <65 | male | T4 | N2 |
| GSM2235927 | ≥65 | male | T4 | N1 |
| GSM2235928 | <65 | female | T4 | N1 |
| GSM2235929 | ≥65 | male | T4 | N2 |
| GSM2235930 | <65 | male | T4 | N2 |
| GSM2235931 | <65 | male | T4 | N1 |
| GSM2235932 | ≥65 | female | T4 | N2 |
| GSM2235933 | <65 | female | T4 | N0 |
| GSM2235934 | <65 | male | T4 | N2 |
| GSM2235935 | ≥65 | male | T4 | N2 |
| GSM2235936 | <65 | female | T1 | N0 |
| GSM2235937 | <65 | female | T4 | N2 |
| GSM2235938 | <65 | female | T1 | N0 |
| GSM2235939 | <65 | female | T2 | N0 |
| GSM2235940 | <65 | male | T4 | N2 |
| GSM2235941 | <65 | female | T2 | N1 |
| GSM2235942 | ≥65 | male | T1 | N0 |
| GSM2235943 | ≥65 | male | T4 | N1 |
| GSM2235944 | <65 | male | T3 | N0 |
| GSM2235945 | <65 | female | T4 | N1 |
| GSM2235946 | <65 | male | T4 | N2 |
| GSM2235947 | <65 | male | T4 | N1 |
| GSM2235948 | <65 | male | T4 | N1 |
| GSM2235949 | <65 | male | T4 | N1 |
| GSM2235950 | <65 | male | T4 | N1 |
| GSM2235951 | <65 | female | T4 | N1 |
| GSM2235952 | <65 | female | T4 | N1 |
| GSM2235953 | <65 | female | T4 | N1 |
| GSM2235954 | <65 | female | T3 | N1 |
| GSM2235955 | <65 | male | T4 | N2 |
| GSM2235956 | ≥65 | female | T4 | N2 |
| GSM2235957 | ≥65 | female | T3 | N2 |
| GSM2235958 | <65 | male | T4 | N2 |
| GSM2235959 | <65 | male | T3 | N2 |
| GSM2235960 | ≥65 | male | T4 | N1 |
| GSM2235961 | <65 | male | T4 | N1 |
| GSM2235962 | <65 | male | T4 | N1 |
| GSM2235963 | <65 | female | T4 | N2 |
| GSM2235964 | <65 | male | T4 | N2 |
| GSM2235965 | <65 | male | T3 | N1 |
| GSM2235966 | <65 | male | T4 | N3 |
| GSM2235967 | <65 | male | T4 | N2 |
| GSM2235968 | <65 | male | T2 | N0 |
| GSM2235969 | ≥65 | female | T2 | N1 |
| GSM2235970 | ≥65 | female | T4 | N2 |
| GSM2235971 | <65 | male | T3 | N2 |
| GSM2235972 | ≥65 | male | T4 | N3 |
| GSM2235973 | <65 | female | T3 | N0 |
| GSM2235974 | <65 | male | T4 | N2 |
| GSM2235975 | <65 | male | T3 | N3 |
| GSM2235976 | ≥65 | female | T3 | N1 |
| GSM2235977 | ≥65 | male | T4 | N2 |
| GSM2235978 | ≥65 | male | T4 | N2 |
| GSM2235979 | <65 | female | T2 | N1 |
| GSM2235980 | ≥65 | male | T4 | N1 |
| GSM2235981 | ≥65 | male | T4 | N1 |
| GSM2235982 | <65 | male | T3 | N2 |
| GSM2235983 | <65 | male | T3 | N1 |
| GSM2235984 | ≥65 | male | T4 | N3 |
| GSM2235985 | ≥65 | female | T4 | N3 |
| GSM2235986 | <65 | female | T3 | N3 |
| GSM2235987 | ≥65 | male | T4 | N1 |
| GSM2235988 | <65 | female | T1 | N0 |
| GSM2235991 | ≥65 | male | T3 | N2 |
| GSM2235992 | <65 | male | T4 | N1 |
| GSM2235993 | <65 | male | T3 | N1 |
| GSM2235994 | <65 | female | T3 | N1 |
| GSM2235995 | <65 | female | T3 | N2 |
| GSM2235996 | <65 | female | T4 | N1 |
| GSM2235997 | ≥65 | male | T4 | N1 |
| GSM2235998 | ≥65 | male | T4 | N1 |
| GSM2235999 | <65 | male | T4 | N1 |
| GSM2236000 | <65 | female | T3 | N1 |
| GSM2236001 | <65 | female | T4 | N2 |
| GSM2236002 | ≥65 | female | T4 | N1 |
| GSM2236003 | <65 | male | T4 | N1 |
| GSM2236004 | ≥65 | male | T4 | N1 |
| GSM2236005 | ≥65 | female | T4 | N2 |
| GSM2236006 | <65 | male | T4 | N1 |
| GSM2236007 | ≥65 | female | T4 | N1 |
| GSM2236008 | <65 | female | T3 | N1 |
| GSM2236009 | ≥65 | male | T4 | N1 |
| GSM2236010 | <65 | male | T4 | N1 |
| GSM2236011 | <65 | male | T3 | N2 |
| GSM2236012 | <65 | male | T4 | N3 |
| GSM2236013 | ≥65 | female | T2 | N1 |
| GSM2236014 | ≥65 | male | T3 | N0 |
| GSM2236015 | ≥65 | male | T4 | N2 |
| GSM2236016 | <65 | male | T4 | N2 |
| GSM2236017 | <65 | male | T4 | N2 |
| GSM2236018 | <65 | male | T4 | N2 |
| GSM2236019 | ≥65 | male | T3 | N2 |
| GSM2236020 | ≥65 | female | T4 | N0 |
| GSM2236021 | <65 | male | T4 | N3 |
| GSM2236022 | <65 | male | T4 | N3 |
| GSM2236023 | <65 | female | T2 | N0 |
| GSM2236024 | <65 | male | T3 | N1 |
| GSM2236025 | ≥65 | female | T3 | N1 |
| GSM2236026 | ≥65 | male | T3 | N1 |
| GSM2236027 | ≥65 | female | T4 | N1 |
| GSM2236028 | ≥65 | male | T3 | N0 |
| GSM2236029 | <65 | male | T2 | N1 |
| GSM2236030 | <65 | male | T4 | N0 |
| GSM2236031 | <65 | female | T4 | N0 |
| GSM2236032 | <65 | male | T4 | N0 |
| GSM2236033 | ≥65 | male | T3 | N0 |
| GSM2236034 | <65 | male | T4 | N0 |
| GSM2236036 | <65 | male | T1 | N1 |
| GSM2236037 | <65 | female | T4 | N1 |
| GSM2236038 | ≥65 | male | T4 | N2 |
| GSM2236039 | <65 | male | T2 | N0 |
| GSM2236040 | ≥65 | female | T3 | N1 |
| GSM2236041 | <65 | male | T4 | N1 |
| GSM2236042 | ≥65 | female | T4 | N2 |
| GSM2236043 | <65 | male | T3 | N1 |
| GSM2236044 | <65 | male | T2 | N2 |
| GSM2236045 | <65 | male | T4 | N1 |
| GSM2236046 | <65 | male | T4 | N1 |
| GSM2236047 | <65 | female | T4 | N2 |
| GSM2236048 | <65 | male | T3 | N0 |
| GSM2236049 | <65 | female | T4 | N1 |
| GSM2236050 | ≥65 | male | T3 | N1 |
| GSM2236051 | <65 | male | T4 | N2 |
| GSM2236052 | <65 | male | T4 | N1 |
| GSM2236053 | <65 | female | T4 | N2 |
| GSM2236054 | <65 | female | T4 | N0 |
| GSM2236055 | <65 | male | T4 | N0 |
| GSM2236056 | <65 | male | T4 | N0 |
| GSM2236057 | <65 | male | T2 | N1 |
| GSM2236058 | <65 | male | T2 | N1 |
| GSM2236059 | ≥65 | female | T2 | N1 |
| GSM2236060 | <65 | male | T4 | N3 |
| GSM2236061 | ≥65 | female | T2 | N1 |
| GSM2236062 | ≥65 | male | T3 | N1 |
| GSM2236063 | <65 | female | T2 | N1 |
| GSM2236064 | ≥65 | male | T4 | N0 |
| GSM2236065 | <65 | male | T4 | N2 |
| GSM2236066 | <65 | female | T4 | N1 |
| GSM2236067 | <65 | male | T4 | N2 |
| GSM2236068 | <65 | male | T4 | N0 |
| GSM2236069 | ≥65 | male | T3 | N1 |
| GSM2236070 | ≥65 | male | T3 | N1 |
| GSM2236071 | <65 | female | T4 | N1 |
| GSM2236072 | <65 | male | T3 | N3 |
| GSM2236073 | <65 | male | T4 | N2 |
| GSM2236074 | <65 | male | T2 | N1 |
| GSM2236075 | <65 | male | T4 | N1 |
| GSM2236076 | <65 | male | T3 | N1 |
| GSM2236077 | ≥65 | male | T4 | N2 |
| GSM2236078 | <65 | male | T4 | N0 |
| GSM2236079 | ≥65 | male | T2 | N1 |
| GSM2236080 | <65 | male | T4 | N0 |
| GSM2236082 | <65 | female | T4 | N0 |
| GSM2236083 | <65 | female | T4 | N2 |
| GSM2236084 | <65 | female | T3 | N2 |
| GSM2236085 | <65 | male | T4 | N0 |
| GSM2236086 | <65 | female | T4 | N0 |
| GSM2236087 | ≥65 | male | T3 | N1 |
| GSM2236088 | <65 | female | T4 | N0 |
| GSM2236089 | ≥65 | female | T4 | N0 |
| GSM2236090 | ≥65 | male | T4 | N0 |
| GSM2236091 | ≥65 | male | T3 | N0 |
| GSM2236092 | <65 | male | T4 | N0 |
| GSM2236093 | <65 | male | T3 | N0 |
| GSM2236094 | <65 | male | T4 | N0 |
| GSM2236095 | <65 | male | T4 | N0 |
