# Supplementary material for: Comprehensive Molecular Analyses of an SLC Family-Based Model in Stomach Adenocarcinoma
Source: Pathol Oncol Res. 2022 Oct 13;28:1610610. doi: 10.3389/pore.2022.1610610 (PMC9606230; doi:10.3389/pore.2022.1610610)
Supplement: Supplementary file 7 [file Table2.DOCX]

**Table S2.**

Multivariate Cox regression analyses of potential prognostic SLC genes in TCGA Cohort.

| **Variables** | ***P* Value** | **HR** | **HR.95L HR.95H** | |
| --- | --- | --- | --- | --- |
| SLC6A9 | 0.007 | 0.783 | 0.619 | 0.990 |
| SLC7A2 | 0.003 | 1.339 | 1.043 | 1.718 |
| SLC7A3 | 0.033 | 1.294 | 0.948 | 1.764 |
| SL25A15 | 0.007 | 0.784 | 0.623 | 0.988 |
