# Supplementary material for: Comprehensive Molecular Analyses of an SLC Family-Based Model in Stomach Adenocarcinoma
Source: Pathol Oncol Res. 2022 Oct 13;28:1610610. doi: 10.3389/pore.2022.1610610 (PMC9606230; doi:10.3389/pore.2022.1610610)
Supplement: Supplementary file 8 [file Table3.DOCX]

**TableS3**

Univariable and multivariable Cox regression analysis of the SLC-based signature and survival in GSE62254.

| **Variable** | univariable analysis | | | |  | multivariable analysis | | | |
| --- | --- | --- | --- | --- | --- | --- | --- | --- | --- |
|  | HR | HR.95L | HR.95H | pvalue |  | HR | HR.95L | HR.95H | pvalue |
| **Age**  ≥65 or <65 | 2.043 | 1.476 | 2.828 | 0.000 |  | 2.059 | 1.490 | 2.845 | 0.000 |
| **Gender**  Male or Female | 1.101 | 0.778 | 1.560 | 0.586 |  |  |  |  |  |
| **TNM Stage**  I or II or III or IV | 2.179 | 1.658 | 2.865 | 0.000 |  | 2.284 | 1.873 | 2.785 | 0.000 |
| **T Stage**  1 or 2 or 3 or 4 | 0.991 | 0.757 | 1.296 | 0.946 |  |  |  |  |  |
| **Hematogenous metastasis**  Yes or no | 1.411 | 0.857 | 2.324 | 0.176 |  |  |  |  |  |
| **Lymphatic metastasis**  Yes or no | 0.979 | 0.470 | 2.037 | 0.954 |  |  |  |  |  |
| **Risk score**  High or Low | 2.213 | 1.579 | 3.101 | 0.000 |  | 2.212 | 1.580 | 3.095 | 0.000 |
