# Supplementary material for: Comprehensive Molecular Analyses of an SLC Family-Based Model in Stomach Adenocarcinoma
Source: Pathol Oncol Res. 2022 Oct 13;28:1610610. doi: 10.3389/pore.2022.1610610 (PMC9606230; doi:10.3389/pore.2022.1610610)
Supplement: Supplementary file 9 [file DataSheet1.docx]

**R project for statistical analysis**

Figure 1AB:

library(survival)

library(survminer)

coxPfilter=0.05

inputFile="TCGA.expTime.txt"

setwd("D:\\AAA Study\\ gastric\\20.uniCox")

rt=read.table(inputFile, header=T, sep="\t", check.names=F, row.names=1)

rt$futime=rt$futime/365

outTab=data.frame()

sigGenes=c("futime","fustat")

for(i in colnames(rt[,3:ncol(rt)])){

cox <- coxph(Surv(futime, fustat) ~ rt[,i], data = rt)

coxSummary = summary(cox)

coxP=coxSummary$coefficients[,"Pr(>|z|)"]

if(coxP<coxPfilter){

sigGenes=c(sigGenes,i)

outTab=rbind(outTab,

cbind(id=i,

HR=coxSummary$conf.int[,"exp(coef)"],

HR.95L=coxSummary$conf.int[,"lower .95"],

HR.95H=coxSummary$conf.int[,"upper .95"],

pvalue=coxSummary$coefficients[,"Pr(>|z|)"])

)

data=rt[,c("futime", "fustat", i)]

colnames(data)=c("futime", "fustat", "gene")

res.cut=surv_cutpoint(data, time = "futime", event = "fustat", variables =c("gene"))

res.cat=surv_categorize(res.cut)

diff=survdiff(Surv(futime, fustat) ~gene,data =res.cat)

pValue=1-pchisq(diff$chisq, df=1)

#print(pValue)

if(pValue<0.001){

pValue="p<0.001"

}else{

pValue=paste0("p=",sprintf("%.03f",pValue))

}

fit=survfit(Surv(futime, fustat) ~gene, data = res.cat)

surPlot=ggsurvplot(fit,

data=res.cat,

pval=pValue,

pval.size=6,

legend.title=i,

legend.labs=c("high","low"),

xlab="Time(years)",

palette=c("red", "blue"),

break.time.by=1,

conf.int=T,

risk.table=TRUE,

risk.table.title="",

risk.table.height=.25)

pdf(file=paste0("survival.",i,".pdf"), onefile=FALSE, width=6, height =5)

print(surPlot)

dev.off()

}

}

write.table(outTab,file="TCGA.uniCox.txt",sep="\t",row.names=F,quote=F)

uniSigExp=rt[,sigGenes]

uniSigExp=cbind(id=row.names(uniSigExp),uniSigExp)

write.table(uniSigExp,file="TCGA.uniSigExp.txt",sep="\t",row.names=F,quote=F)

bioForest=function(coxFile=null,forestFile=null,forestCol=null){

rt <- read.table(coxFile,header=T,sep="\t",row.names=1,check.names=F)

gene <- rownames(rt)

hr <- sprintf("%.3f",rt$"HR")

hrLow <- sprintf("%.3f",rt$"HR.95L")

hrHigh <- sprintf("%.3f",rt$"HR.95H")

Hazard.ratio <- paste0(hr,"(",hrLow,"-",hrHigh,")")

pVal <- ifelse(rt$pvalue<0.001, "<0.001", sprintf("%.3f", rt$pvalue))

height=nrow(rt)/12.5+5

pdf(file=forestFile, width = 7,height = height)

n <- nrow(rt)

nRow <- n+1

ylim <- c(1,nRow)

layout(matrix(c(1,2),nc=2),width=c(3,2.5))

xlim = c(0,3)

par(mar=c(4,2.5,2,1))

plot(1,xlim=xlim,ylim=ylim,type="n",axes=F,xlab="",ylab="")

text.cex=0.8

text(0,n:1,gene,adj=0,cex=text.cex)

text(1.5-0.5*0.2,n:1,pVal,adj=1,cex=text.cex);text(1.5-0.5*0.2,n+1,'pvalue',cex=text.cex,font=2,adj=1)

text(3,n:1,Hazard.ratio,adj=1,cex=text.cex);text(3,n+1,'Hazard ratio',cex=text.cex,font=2,adj=1,)

par(mar=c(4,1,2,1),mgp=c(2,0.5,0))

xlim = c(0,max(as.numeric(hrLow),as.numeric(hrHigh)))

plot(1,xlim=xlim,ylim=ylim,type="n",axes=F,ylab="",xaxs="i",xlab="Hazard ratio")

arrows(as.numeric(hrLow),n:1,as.numeric(hrHigh),n:1,angle=90,code=3,length=0.05,col="darkblue",lwd=2.5)

abline(v=1,col="black",lty=2,lwd=2)

boxcolor = ifelse(as.numeric(hr) > 1, forestCol[1], forestCol[2])

points(as.numeric(hr), n:1, pch = 15, col = boxcolor, cex=1.6)

axis(1)

dev.off()

}

bioForest(coxFile="TCGA.uniCox.txt",forestFile="forest.pdf",forestCol=c("red","green"))

Figure 1C:

library(reshape2)

library(ggpubr)

library(ggExtra)

library(pheatmap)

bioRiskPlot=function(inputFile=null, riskScoreFile=null, survStatFile=null, survCorFile=null){

rt=read.table(inputFile, header=T, sep="\t", check.names=F, row.names=1)

rt$riskScore[rt$riskScore>quantile(rt$riskScore,0.99)]=quantile(rt$riskScore,0.99)

rt$risk=factor(rt$risk, levels=c("low", "high"))

rt=rt[order(rt$riskScore),]

riskClass=rt[,"Risk"]

lowLength=length(riskClass[riskClass=="low"])

highLength=length(riskClass[riskClass=="high"])

lowMax=max(rt$riskScore[riskClass=="low"])

line=rt[,"riskScore"]

pdf(file=riskScoreFile, width=6, height=4)

plot(line, type="p", pch=20,

xlab="Patients (increasing risk socre)", ylab="Risk score",

col=c(rep("blue",lowLength),rep("red",highLength)) )

abline(h=lowMax,v=lowLength,lty=2)

legend("topleft", c("High risk", "Low Risk"),bty="n",pch=19,col=c("red","blue"),cex=1.2)

dev.off()

color=as.vector(rt$fustat)

color[color==1]="red"

color[color==0]="blue"

pdf(file=survStatFile, width=6, height=4)

plot(rt$futime, pch=19,

xlab="Patients (increasing risk socre)", ylab="Survival time (years)",

col=color)

legend("topleft", c("Dead", "Alive"),bty="n",pch=19,col=c("red","blue"),cex=1.2)

abline(v=lowLength,lty=2)

dev.off()

x=as.numeric(rt[,"riskScore"])

y=as.numeric(rt[,"futime"])

df1=as.data.frame(cbind(x,y))

p1=ggplot(df1, aes(x, y)) +

xlab("Risk score") +

ylab("OS (years)") +

geom_point() + geom_smooth(method="lm",formula = y ~ x) + theme_bw()+

stat_cor(method = 'spearman', aes(x =x, y =y))

p2=ggMarginal(p1, type="density", xparams=list(fill = "orange"), yparams=list(fill = "blue"))

pdf(file=survCorFile, width=5.2, height=5)

print(p2)

dev.off()

}

bioRiskPlot(inputFile="TCGA.txt",

riskScoreFile="train.riskScore.pdf",

survStatFile="train.survStat.pdf",

survCorFile="train.survCor.pdf")

Figure 1D:

library(survival)

library(survminer)

setwd("D:\\AAA Study\\gastric\\23.survival")

bioSurvival=function(inputFile=null,outFile=null){

rt=read.table(inputFile, header=T, sep="\t", check.names=F)

diff=survdiff(Surv(futime, fustat) ~ Risk,data = rt)

pValue=1-pchisq(diff$chisq,df=1)

if(pValue<0.001){

pValue="p<0.001"

}else{

pValue=paste0("p=",sprintf("%.03f",pValue))

}

fit <- survfit(Surv(futime, fustat) ~ Risk, data = rt)

surPlot=ggsurvplot(fit,

data=rt,

conf.int=T,

pval=pValue,

pval.size=6,

legend.title="Risk",

legend.labs=c("High risk", "Low risk"),

xlab="Time(years)",

break.time.by = 1,

palette=c("red", "blue"),

risk.table=TRUE,

risk.table.title="",

risk.table.height=.25)

pdf(file=outFile,onefile = FALSE,width = 6.5,height =5.5)

print(surPlot)

dev.off()

}

bioSurvival(inputFile="TCGA.txt", outFile="survival.TCGA.pdf")

Figure 2AC:

library(reshape2)

library(ggpubr)

library(ggExtra)

library(pheatmap)

bioRiskPlot=function(inputFile=null, riskScoreFile=null, survStatFile=null, survCorFile=null){

rt=read.table(inputFile, header=T, sep="\t", check.names=F, row.names=1)

rt$riskScore[rt$riskScore>quantile(rt$riskScore,0.99)]=quantile(rt$riskScore,0.99)

rt$risk=factor(rt$risk, levels=c("low", "high"))

rt=rt[order(rt$riskScore),]

riskClass=rt[,"Risk"]

lowLength=length(riskClass[riskClass=="low"])

highLength=length(riskClass[riskClass=="high"])

lowMax=max(rt$riskScore[riskClass=="low"])

line=rt[,"riskScore"]

pdf(file=riskScoreFile, width=6, height=4)

plot(line, type="p", pch=20,

xlab="Patients (increasing risk socre)", ylab="Risk score",

col=c(rep("blue",lowLength),rep("red",highLength)) )

abline(h=lowMax,v=lowLength,lty=2)

legend("topleft", c("High risk", "Low Risk"),bty="n",pch=19,col=c("red","blue"),cex=1.2)

dev.off()

color=as.vector(rt$fustat)

color[color==1]="red"

color[color==0]="blue"

pdf(file=survStatFile, width=6, height=4)

plot(rt$futime, pch=19,

xlab="Patients (increasing risk socre)", ylab="Survival time (years)",

col=color)

legend("topleft", c("Dead", "Alive"),bty="n",pch=19,col=c("red","blue"),cex=1.2)

abline(v=lowLength,lty=2)

dev.off()

x=as.numeric(rt[,"riskScore"])

y=as.numeric(rt[,"futime"])

df1=as.data.frame(cbind(x,y))

p1=ggplot(df1, aes(x, y)) +

xlab("Risk score") +

ylab("OS (years)") +

geom_point() + geom_smooth(method="lm",formula = y ~ x) + theme_bw()+

stat_cor(method = 'spearman', aes(x =x, y =y))

p2=ggMarginal(p1, type="density", xparams=list(fill = "orange"), yparams=list(fill = "blue"))

pdf(file=survCorFile, width=5.2, height=5)

print(p2)

dev.off()

}

bioRiskPlot(inputFile="GSE.txt",

riskScoreFile="train.riskScore.pdf",

survStatFile="train.survStat.pdf",

survCorFile="train.survCor.pdf")

Figure 2BD:

library(survival)

library(survminer)

setwd("D:\\AAA Study\\gastric\\23.survival")

bioSurvival=function(inputFile=null,outFile=null){

rt=read.table(inputFile, header=T, sep="\t", check.names=F)

diff=survdiff(Surv(futime, fustat) ~ Risk,data = rt)

pValue=1-pchisq(diff$chisq,df=1)

if(pValue<0.001){

pValue="p<0.001"

}else{

pValue=paste0("p=",sprintf("%.03f",pValue))

}

fit <- survfit(Surv(futime, fustat) ~ Risk, data = rt)

surPlot=ggsurvplot(fit,

data=rt,

conf.int=T,

pval=pValue,

pval.size=6,

legend.title="Risk",

legend.labs=c("High risk", "Low risk"),

xlab="Time(years)",

break.time.by = 1,

palette=c("red", "blue"),

risk.table=TRUE,

risk.table.title="",

risk.table.height=.25)

pdf(file=outFile,onefile = FALSE,width = 6.5,height =5.5)

print(surPlot)

dev.off()

}

bioSurvival(inputFile="GSE.txt", outFile="survival.GEO.pdf")

Figure 3A:

library(ComplexHeatmap)

riskFile="TCGA.txt"

cliFile="TCGAcli.txt"

setwd("D:\\AAA Study\\gastric\\35.cliHeatmap")

risk=read.table(riskFile, header=T, sep="\t", check.names=F, row.names=1)

risk=risk[order(risk$riskScore),]

cli=read.table(cliFile,sep="\t",header=T,check.names=F,row.names=1)

samSample=intersect(row.names(risk), row.names(cli))

risk=risk[samSample,"Risk",drop=F]

cli=cli[samSample,,drop=F]

rt=cbind(cli, risk)

sigVec=c()

for(clinical in colnames(rt[,1:(ncol(rt)-1)])){

data=rt[c("Risk", clinical)]

colnames(data)=c("Risk", "clinical")

data=data[(data[,"clinical"]!="unknow"),]

tableStat=table(data)

stat=chisq.test(tableStat)

pvalue=stat$p.value

Sig=ifelse(pvalue<0.001,"***",ifelse(pvalue<0.01,"**",ifelse(pvalue<0.05,"*","")))

sigVec=c(sigVec, paste0(clinical, Sig))

}

sigVec=c(sigVec, "Risk")

colnames(rt)=sigVec

rt$Risk=factor(rt$Risk, levels=c("low","high"))

bioCol=c("#0066FF","#FF9900","#FF0000","#ed1299", "#0dbc21", "#246b93", "#cc8e12", "#d561dd", "#c93f00",

"#ce2523", "#f7aa5d", "#9ed84e", "#39ba30", "#6ad157", "#373bbf", "#a1ce4c", "#ef3bb6", "#d66551",

"#1a918f", "#7149af", "#ff66fc", "#2927c4", "#57e559" ,"#8e3af4" ,"#f9a270" ,"#22547f", "#db5e92",

"#4aef7b", "#e86502", "#99db27", "#e07233", "#8249aa","#cebb10", "#03827f", "#931635", "#ff523f",

"#edd05e", "#6f25e8", "#0dbc21", "#167275", "#280f7a", "#6373ed", "#5b910f" ,"#7b34c1" ,"#0cf29a" ,"#d80fc1",

"#dd27ce", "#07a301", "#ddd53e", "#391c82", "#2baeb5","#925bea", "#09f9f5", "#63ff4f")

colorList=list()

j=0

for(cli in colnames(rt[,1:(ncol(rt)-1)])){

cliLength=length(levels(factor(rt[,cli])))

cliCol=bioCol[(j+1):(j+cliLength)]

j=j+cliLength

names(cliCol)=levels(factor(rt[,cli]))

cliCol["unknow"]="grey75"

colorList[[cli]]=cliCol

}

colorList[["Risk"]]=c("low"="blue", "high"="red")

ha=HeatmapAnnotation(df=rt, col=colorList)

zero_row_mat=matrix(nrow=0, ncol=nrow(rt))

Hm=Heatmap(zero_row_mat, top_annotation=ha)

pdf(file="heatmap.pdf", width=7, height=5)

draw(Hm, merge_legend=TRUE, heatmap_legend_side="bottom", annotation_legend_side="bottom")

dev.off()

Figure 3B:

library(maftools)

setwd("C:\\biowolf\\IRGPI\\27.riskMaftools")

risk=read.table("TCGA.txt", header=T, sep="\t", check.names=F)

outTab=risk[,c(1, ncol(risk))]

colnames(outTab)=c("Tumor_Sample_Barcode", "Risk")

write.table(outTab, file="ann.txt", sep="\t", quote=F, row.names=F)

geneNum=20

geneMut=read.table("geneMut.txt", header=T, sep="\t", check.names=F, row.names=1)

gene=row.names(geneMut)[1:geneNum]

ann_colors=list()

col=c("blue", "red")

names(col)=c("low", "high")

ann_colors[["Risk"]]=col

pdf(file="low.pdf", width=6, height=6)

maf=read.maf(maf="low.maf", clinicalData="ann.txt")

oncoplot(maf=maf, clinicalFeatures="Risk", genes=gene, annotationColor=ann_colors, keepGeneOrder=T)

dev.off()

pdf(file="high.pdf", width=6, height=6)

maf=read.maf(maf="high.maf", clinicalData="ann.txt")

oncoplot(maf=maf, clinicalFeatures="Risk", genes=gene, annotationColor=ann_colors, keepGeneOrder=T)

dev.off()

Figure 3C:

library(limma)

library(org.Hs.eg.db)

library(clusterProfiler)

library(enrichplot)

expFile="TCGAexp.txt"

riskFile="TCGA.txt"

gmtFile="c2.cp.kegg.v7.4.symbols.gmt"

rt=read.table(expFile, header=T, sep="\t", check.names=F)

rt=as.matrix(rt)

rownames(rt)=rt[,1]

exp=rt[,2:ncol(rt)]

dimnames=list(rownames(exp),colnames(exp))

data=matrix(as.numeric(as.matrix(exp)),nrow=nrow(exp),dimnames=dimnames)

data=avereps(data)

data=data[rowMeans(data)>0.5,]

group=sapply(strsplit(colnames(data),"\\-"), "[", 4)

group=sapply(strsplit(group,""), "[", 1)

group=gsub("2", "1", group)

data=data[,group==0]

data=t(data)

rownames(data)=gsub("(.*?)\\-(.*?)\\-(.*?)\\-.*", "\\1\\-\\2\\-\\3", rownames(data))

data=t(avereps(data))

Risk=read.table(riskFile, header=T, sep="\t", check.names=F, row.names=1)

data=data[,row.names(Risk)]

dataL=data[,row.names(Risk[Risk[,"Risk"]=="low",])]

dataH=data[,row.names(Risk[Risk[,"Risk"]=="high",])]

meanL=rowMeans(dataL)

meanH=rowMeans(dataH)

meanL[meanL<0.00001]=0.00001

meanH[meanH<0.00001]=0.00001

logFC=log2(meanH)-log2(meanL)

logFC=sort(logFC,decreasing=T)

genes=names(logFC)

gmt=read.gmt(gmtFile)

kk=GSEA(logFC, TERM2GENE=gmt, pvalueCutoff = 1)

kkTab=as.data.frame(kk)

kkTab=kkTab[kkTab$pvalue<0.05,]

write.table(kkTab,file="GSEA.result.txt",sep="\t",quote=F,row.names = F)

termNum=5

kkUp=kkTab[kkTab$NES>0,]

if(nrow(kkUp)>=termNum){

showTerm=row.names(kkUp)[1:termNum]

gseaplot=gseaplot2(kk, showTerm, base_size=8, title="Enriched in high risk group")

pdf(file="GSEA.highRisk.pdf", width=7, height=5.5)

print(gseaplot)

dev.off()

}

termNum=5

kkDown=kkTab[kkTab$NES<0,]

if(nrow(kkDown)>=termNum){

showTerm=row.names(kkDown)[1:termNum]

gseaplot=gseaplot2(kk, showTerm, base_size=8, title="Enriched in low risk group")

pdf(file="GSEA.lowRisk.pdf", width=7, height=5.5)

print(gseaplot)

dev.off()

}

Figure 4:

library("clusterProfiler")

library("org.Hs.eg.db")

library("enrichplot")

library("ggplot2")

library(GOplot)

pvalueFilter=0.05

qvalueFilter=0.05

colorSel="qvalue"

if(qvalueFilter>0.05){

colorSel="pvalue"

}

setwd("C:\\biowolf\\IRGPI\\14.KEGG")

rt=read.table("diff.txt", header=T, sep="\t", check.names=F)

genes=as.vector(rt[,1])

entrezIDs=mget(genes, org.Hs.egSYMBOL2EG, ifnotfound=NA)

entrezIDs=as.character(entrezIDs)

rt=cbind(rt,entrezID=entrezIDs)

gene=entrezIDs[entrezIDs!="NA"]

kk <- enrichKEGG(gene=gene, organism="hsa", pvalueCutoff=1, qvalueCutoff=1)

KEGG=as.data.frame(kk)

KEGG$geneID=as.character(sapply(KEGG$geneID,function(x)paste(rt$gene[match(strsplit(x,"/")[[1]],as.character(rt$entrezID))],collapse="/")))

KEGG=KEGG[(KEGG$pvalue<pvalueFilter & KEGG$qvalue<qvalueFilter),]

write.table(KEGG, file="KEGG.txt", sep="\t", quote=F, row.names = F)

showNum=30

if(nrow(KEGG)<showNum){

showNum=nrow(KEGG)

}

pdf(file="barplot.pdf", width=8, height=7)

barplot(kk, drop = TRUE, showCategory = showNum, color = colorSel)

dev.off()

pdf(file="bubble.pdf", width=8, height=7)

dotplot(kk, showCategory = showNum, orderBy = "GeneRatio",color = colorSel)

dev.off()

kegg=data.frame(Category="ALL", ID = KEGG$ID, Term=KEGG$Description, Genes = gsub("/", ", ", KEGG$geneID), adj_pval = KEGG$p.adjust)

genelist <- data.frame(ID = rt$gene, logFC = rt$logFC)

row.names(genelist)=genelist[,1]

circ <- circle_dat(kegg, genelist)

termNum =8

termNum=ifelse(nrow(kegg)<termNum,nrow(kegg),termNum)

geneNum=200

geneNum=ifelse(nrow(genelist)<geneNum, nrow(genelist), geneNum)

chord <- chord_dat(circ, genelist[1:geneNum,], kegg$Term[1:termNum])

pdf(file="KEGGcircos.pdf", width=11, height=11)

GOChord(chord,

space = 0.001,

gene.order = 'logFC',

gene.space = 0.25,

gene.size = 5,

border.size = 0.1,

process.label = 6)

dev.off()

Figure 5ABC:

library(limma)

library(reshape2)

library(ggpubr)

riskFile="input.txt"

immFile="CIBERSORT-Results.txt"

pFilter=0.05

setwd("D:\\AAA Study\\gastric\\29.immCor")

immune=read.table(immFile, header=T, sep="\t", check.names=F, row.names=1)

immune=immune[immune[,"P-value"]<pFilter,]

data=as.matrix(immune[,1:(ncol(immune)-3)])

group=sapply(strsplit(row.names(data),"\\-"), "[", 4)

group=sapply(strsplit(group,""), "[", 1)

group=gsub("2", "1", group)

data=data[group==0,]

row.names(data)=gsub("(.*?)\\-(.*?)\\-(.*?)\\-(.*?)\\-.*", "\\1\\-\\2\\-\\3", row.names(data))

data=avereps(data)

risk=read.table(riskFile, header=T, sep="\t", check.names=F, row.names=1)

sameSample=intersect(row.names(data), row.names(risk))

rt=cbind(data[sameSample,,drop=F], risk[sameSample,"Risk",drop=F])

rt=rt[order(rt$Risk, decreasing=T),]

conNum=nrow(rt[rt$Risk=="low",])

treatNum=nrow(rt[rt$Risk=="high",])

data=t(rt[,-ncol(rt)])

pdf("barplot.pdf", height=10, width=18)

col=rainbow(nrow(data), s=0.7, v=0.7)

par(las=1,mar=c(8,5,4,16),mgp=c(3,0.1,0),cex.axis=1.5)

a1=barplot(data,col=col,yaxt="n",ylab="Relative Percent",xaxt="n",cex.lab=1.8)

a2=axis(2,tick=F,labels=F)

axis(2,a2,paste0(a2*100,"%"))

par(srt=0,xpd=T)

rect(xleft = a1[1], ybottom = -0.01, xright = a1[conNum], ytop = -0.06,col="green")

text(a1[conNum]/2,-0.035,"Low risk",cex=2)

rect(xleft = a1[conNum], ybottom = -0.01, xright =a1[length(a1)] , ytop = -0.06,col="red")

text((a1[length(a1)]+a1[conNum])/2,-0.035,"High risk",cex=2)

ytick2 = cumsum(data[,ncol(data)])

ytick1 = c(0,ytick2[-length(ytick2)])

legend(par('usr')[2]*0.98,par('usr')[4],legend=rownames(data),col=col,pch=15,bty="n",cex=1.3)

dev.off()

data=rt

data=melt(data, id.vars=c("Risk"))

colnames(data)=c("Risk", "Immune", "Expression")

group=levels(factor(data$Risk))

data$Risk=factor(data$Risk, levels=c("low","high"))

bioCol=c("#0066FF","#FF0000","#6E568C","#7CC767","#223D6C","#D20A13","#FFD121","#088247","#11AA4D")

bioCol=bioCol[1:length(group)]

boxplot=ggboxplot(data, x="Immune", y="Expression", fill="Risk",

xlab="",

ylab="Fraction",

legend.title="Risk",

width=0.8,

palette=bioCol)+

rotate_x_text(50)+

stat_compare_means(aes(group=Risk),symnum.args=list(cutpoints=c(0, 0.001, 0.01, 0.05, 1), symbols=c("***", "**", "*", "")), label="p.signif")

pdf(file="immune.diff.pdf", width=8, height=6)

print(boxplot)

dev.off()

Figure 5D:

library(limma)

library(GSVA)

library(GSEABase)

library(ggpubr)

library(reshape2)

expFile="symbol.txt"

gmtFile="immune.gmt"

riskFile="risk.TCGA.txt"

setwd("D:\\AAA Study\\gastric\\31.immFunction")

rt=read.table(expFile, header=T, sep="\t", check.names=F)

rt=as.matrix(rt)

rownames(rt)=rt[,1]

exp=rt[,2:ncol(rt)]

dimnames=list(rownames(exp),colnames(exp))

mat=matrix(as.numeric(as.matrix(exp)),nrow=nrow(exp),dimnames=dimnames)

mat=avereps(mat)

mat=mat[rowMeans(mat)>0,]

geneSet=getGmt(gmtFile, geneIdType=SymbolIdentifier())

ssgseaScore=gsva(mat, geneSet, method='ssgsea', kcdf='Gaussian', abs.ranking=TRUE)

normalize=function(x){

return((x-min(x))/(max(x)-min(x)))}

data=normalize(ssgseaScore)

ssgseaOut=rbind(id=colnames(data), data)

write.table(ssgseaOut, file="immFunScore.txt", sep="\t", quote=F, col.names=F)

group=sapply(strsplit(colnames(data),"\\-"), "[", 4)

group=sapply(strsplit(group,""), "[", 1)

group=gsub("2", "1", group)

data=t(data[,group==0])

rownames(data)=gsub("(.*?)\\-(.*?)\\-(.*?)\\-(.*?)\\-.*", "\\1\\-\\2\\-\\3", rownames(data))

data=avereps(data)

risk=read.table(riskFile, header=T, sep="\t", check.names=F, row.names=1)

sameSample=intersect(row.names(data),row.names(risk))

data=data[sameSample,,drop=F]

risk=risk[sameSample,"Risk",drop=F]

rt1=cbind(data, risk)

data=melt(rt1, id.vars=c("Risk"))

colnames(data)=c("Risk","Type","Score")

data$Risk=factor(data$Risk, levels=c("low","high"))

p=ggboxplot(data, x="Type", y="Score", color = "Risk",

xlab="",ylab="Score",add = "none",palette = c("blue","red") )

p=p+rotate_x_text(50)

p=p+stat_compare_means(aes(group=Risk),symnum.args=list(cutpoints = c(0, 0.001, 0.01, 0.05, 1), symbols = c("***", "**", "*", "")),label = "p.signif")

pdf(file="immFunction.pdf", width=8, height=6)

print(p)

dev.off()
